# Subtle shifts in microbial communities occur alongside the release of carbon induced by drought and rewetting in contrasting peatland ecosystems

**DOI:** 10.1038/s41598-017-11546-w

**Published:** 2017-09-12

**Authors:** Caitlin Potter, Chris Freeman, Peter N. Golyshin, Gail Ackermann, Nathalie Fenner, James E. McDonald, Abdassalam Ehbair, Timothy G. Jones, Loretta M. Murphy, Simon Creer

**Affiliations:** 1 School of Biological Sciences, Bangor University, Gwynedd, Wales UK; 2 BioFrontiers Institute, University of Colorado at Boulder, Boulder, USA; 3 School of Chemistry, Bangor University, Gwynedd, Wales UK

## Abstract

Peat represents a globally significant pool of sequestered carbon. However, peatland carbon stocks are highly threatened by anthropogenic climate change, including drought, which leads to a large release of carbon dioxide. Although the enzymatic mechanisms underlying drought-driven carbon release are well documented, the effect of drought on peatland microbial communities has been little studied. Here, we carried out a replicated and controlled drought manipulation using intact peat ‘mesocosm cores’ taken from bog and fen habitats, and used a combination of community fingerprinting and sequencing of marker genes to identify community changes associated with drought. Community composition varied with habitat and depth. Moreover, community differences between mesocosm cores were stronger than the effect of the drought treatment, emphasising the importance of replication in microbial marker gene studies. While the effect of drought on the overall composition of prokaryotic and eukaryotic communities was weak, a subset of the microbial community did change in relative abundance, especially in the fen habitat at 5 cm depth. ‘Drought-responsive’ OTUs were disproportionately drawn from the phyla *Bacteroidetes* and *Proteobacteria*. Collectively, the data provide insights into the microbial community changes occurring alongside drought-driven carbon release from peatlands, and suggest a number of novel avenues for future research.

## Introduction

Anthropogenic climate change is one of the key issues of the 21^st^ century, with the potential to severely impact human lives as well as natural ecosystems^1^. The effect of climate change on soil biodiversity and consequent ecological processes is of particular concern because of the potential for detrimental positive feedback effects. An overarching concern is that warming can lead to an increase in soil respiration and consequently an increased release of carbon dioxide into the atmosphere^2–5^. Micro-organisms should be considered when attempting to understand and predict the effects of climate change on soil processes, since microbial communities are central to the decomposition of soil organic matter^6^ and are directly responsible for a large proportion of soil respiration^7^. In addition, microbial communities play a key role in determining gas fluxes^8^ and rates of nutrient cycling^9^. While it is difficult to separate effects which are mediated by changes to soil microbial communities from the direct effects of environmental change, there is strong evidence that the soil microbial community is important in determining the way that soil processes respond to environmental change^10–12^.

Peat soils are an extremely important global store of carbon: estimates for the total amount of carbon stored in Northern peatlands vary from 273 Gt C to 547 Gt C^13, 14^. However, climate change represents a serious threat to temperate peatlands; for example, within the UK the area covered by blanket peat is projected to decline, with the potential for peatlands to change from carbon sinks to carbon sources^15^. Likewise, the amount of carbon stored within peatlands in Canada^16^, the USA^17^ and across the Northern hemisphere^18^ is also predicted to decline.

The effect of drought on carbon cycling within peatlands has been an area of particular interest. While climate change models project an increase in total precipitation at high latitudes, rainfall is likely to become more concentrated in extreme events interspersed with periods of dry weather^19^, while higher temperatures will increase water loss from soils^20^. Together, these effects will lead to an increase in the likelihood of drought events^19, 20^ and a fall in peatland summer water table^21, 22^. Unlike drier habitats, where drought leads to moisture-limiting conditions and a reduction in carbon release by heterotrophic micro-organisms^6^, drought in peatlands facilitates the aeration of previously anaerobic peat layers. Aeration stimulates microbial decomposition, and consequently leads to increased carbon dioxide release^23^. The effect of drought on peatland carbon dioxide fluxes often outlasts the duration of the drought itself by a considerable margin due to the degradation of inhibitory phenolic compounds under anaerobic conditions^23^. Therefore, the effects of increased summer drought frequency on peatland carbon fluxes represent a potential positive feedback loop, with the potential to accelerate rates of global warming. There is some evidence that the composition of peatland microbial communities responds to long-term water table changes^24–26^, with Actinobacteria and fungi responding particularly strongly after several years of water table drawdown^24^. Microbial community composition also changes in response to short-term drought^27–29^, although the exact microbial groups involved remain unclear. Moreover, while protozoa have been neglected in modern-day studies of drought effects on peatland microbial communities, paleoecological studies indicate that testate amoebae community composition in peat is strongly influenced by water table depth^30^. Nevertheless, the microbial mechanisms underlying drought-driven carbon dioxide release from peat remain poorly understood.

The development of high-throughput sequencing-based approaches for the identification of microorganisms has provided an unprecedented opportunity to advance our understanding of microbial communities in natural environments, and to explore the effects of environmental change on these communities. Initial DNA-based microbial ecology studies were limited by the low throughput of existing sequencing methodologies or the low resolution of ‘community fingerprinting’ approaches^31^, but the introduction of high-throughput sequencing platforms immediately decreased the cost per base pair of sequencing data. Lower sequencing costs and paradigm shifts in throughput have enabled sequencing of rRNA genes to be used on much broader scales and across a wide spectrum of biological diversity^32^.

**Figure 1:**
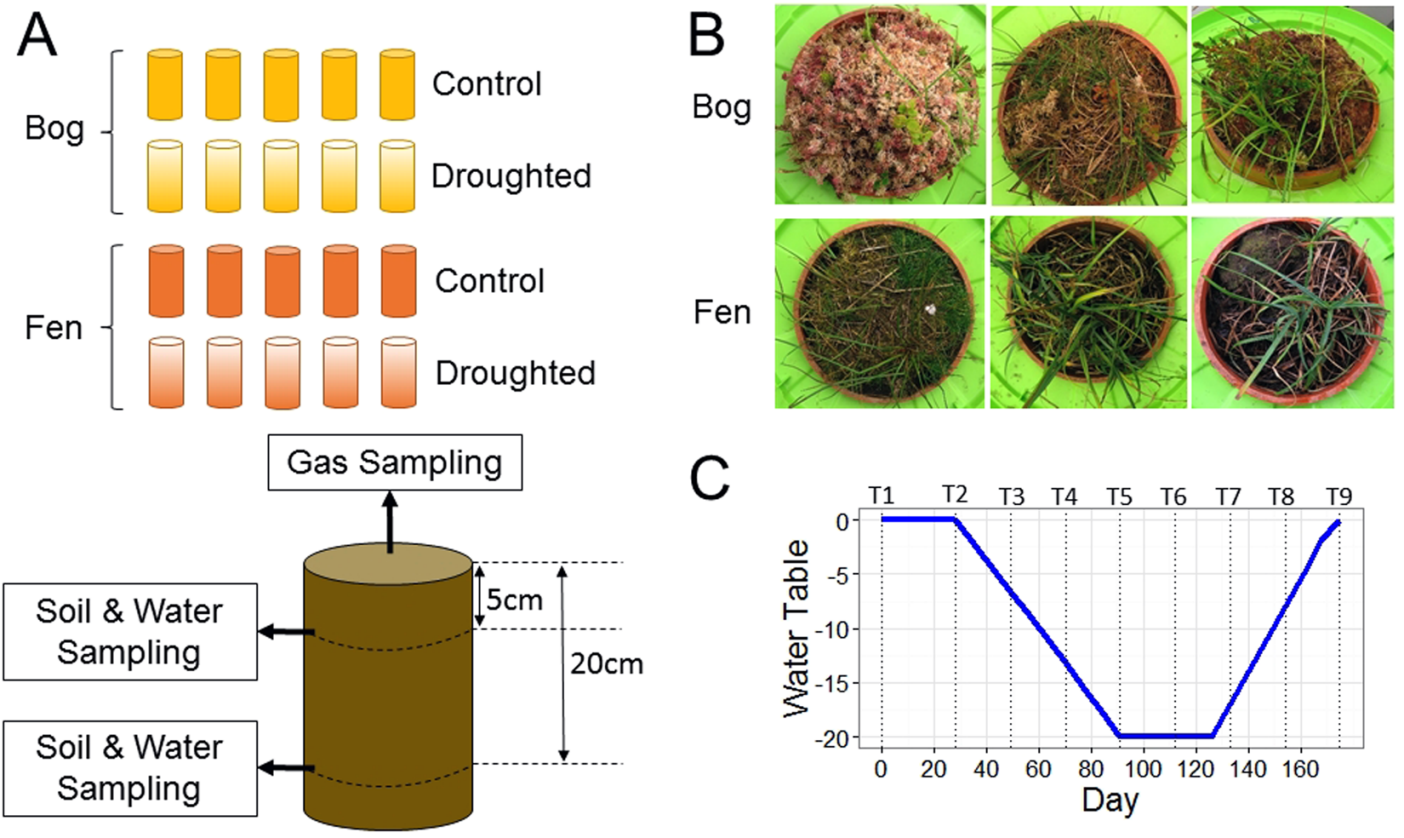
Schematic showing aspects of the experimental design. (**A**) Overview of the experiment showing the number of mesocosm cores collected for each treatment and habitat, as well as depths at which sample collection was carried out. (**B**) View of mesocosm cores from above, giving an indication of typical vegetation in bog and fen. (**C**) Position of the water table throughout the experiment, with sampling time points indicated by dotted lines. Biogeochemical measurements (including measurement of gas fluxes) were carried out on samples taken from all mesocosm cores, but it was only possible to analyse DNA from a randomly selected subsample of three mesocosm cores within each combination of treatment and habitat.

In order to identify changes in microbial communities which occur concurrently to the release of carbon from peat ecosystems, here we aimed to use high-throughput marker gene sequencing to identify the proportion of the microbial biosphere which is affected by drought and rewetting in bogs and fens, two habitats which are representative of the majority of temperate peatlands in the Northern hemisphere. A replicated and controlled drought manipulation was carried out using peat ‘mesocosm cores’ collected from both habitats (Fig. 1). In addition to the concurrent monitoring of greenhouse gas fluxes, DNA was extracted and purified from two contrasting depths below the peat surface. Extracted DNA was then subjected to automated ribosomal intergenic spacer analysis (ARISA), a community fingerprinting technique enabling rapid and low-cost estimation of diversity within a microbial community, in order to confirm that drought affected microbial communities. ARISA fingerprinting was followed by sequencing, bioinformatics and statistical analysis of 16S and 18S rRNA genes to obtain a more detailed perspective of community changes.

## Results

ARISA fingerprinting of bacterial communities yielded bands ranging in size from 110–2839 bp, while ARISA fingerprinting of fungal communities yielded bands ranging from 54–2851 bp. Binning of ARISA amplicons into 5 bp bins gave a total of 185 bins for bacterial communities and 87 for fungal communities.

Sequencing yielded a total of 102,439,895 and 104,156,662 paired-end reads for 16S and 18S rRNA genes, respectively. Of the 16S rRNA gene reads, 29,337,117 passed quality control steps and were clustered into 49,892 OTUs. Of the 18S rRNA gene reads, a total of 17,214,346 passed quality control and paired-end joining, which were clustered into 43,058 OTUs. Following standardisation of read numbers, rarefaction curves were generated to assess sequencing coverage (Fig. S1) and these suggested that sequencing coverage was adequate, particularly for samples from the bog.

### Effects of Habitat and Depth on Microbial Community

**Figure 2:**
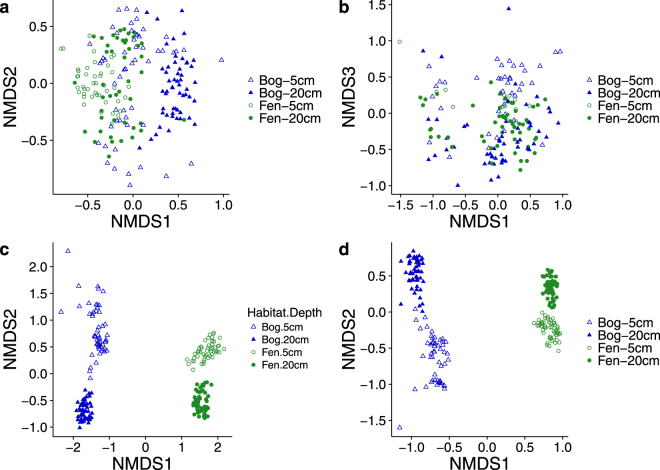
NMDS ordination of microbial communities based on ARISA fingerprinting profiles from (**a**) bacterial and (**b**) fungal communities, and sequencing-based community composition of (**c**) prokaryotes and (**d**) eukaryotes. NMDS ordination was based on Jaccard distances for ARISA data, and Bray-Curtis dissimilarities for sequencing data. OTUs assigned to the following phyla were excluded from the 18S rRNA sequencing dataset prior to ordination: Holozoa, Metazoa, Chloroplastida and ‘NA’. Note that (**b**) shows the first and third axes as the strongest habitat effect occurred along the third axis while other plots display the first and second axes.

NMDS ordination of ARISA fingerprinting data showed some separation of samples taken from bog mesocosm cores at 20 cm depth from other habitats and depths (Fig. 2a). Fungal communities were more weakly affected by habitat and depth, but samples taken from the bog at 5 cm appeared to be distinct from all other samples on the third axis (Fig. 2b). PERMANOVA tests confirmed that ARISA fingerprinting profiles of both bacterial and fungal communities were significantly affected by habitat (bacteria: *P* = 0.001; fungi: *P* = 0.001; Table S1) and depth (bacteria: *P* = 0.001; fungi: *P* = 0.001; Table S1) although in each case the effect size (R^2^ value) was small (Table S1), indicating that habitat and depth only accounted for a small proportion of overall variation. Bacterial communities were also significantly affected by the interaction between habitat and depth (*P* = 0.001; Table S1).

Sequencing of 16S and 18S rRNA genes identified an effect of habitat and depth on both prokaryotic (16S) and eukaryotic (18S) communities that was stronger than that detected by ARISA fingerprinting. For both markers, samples clustered by habitat along the first axis and by depth along the second axis (Fig. 2c,d). PERMANOVA tests confirmed that there were significant effects of habitat (16S: *P* = 0.001; 18S: *P* = 0.001; Table S2), depth (16S: *P* = 0.001; 18S: *P* = 0.001; Table S2) and the interaction term (16S: *P* = 0.001; 18S: *P* = 0.001; Table S2) on community composition.

**Figure 3:**
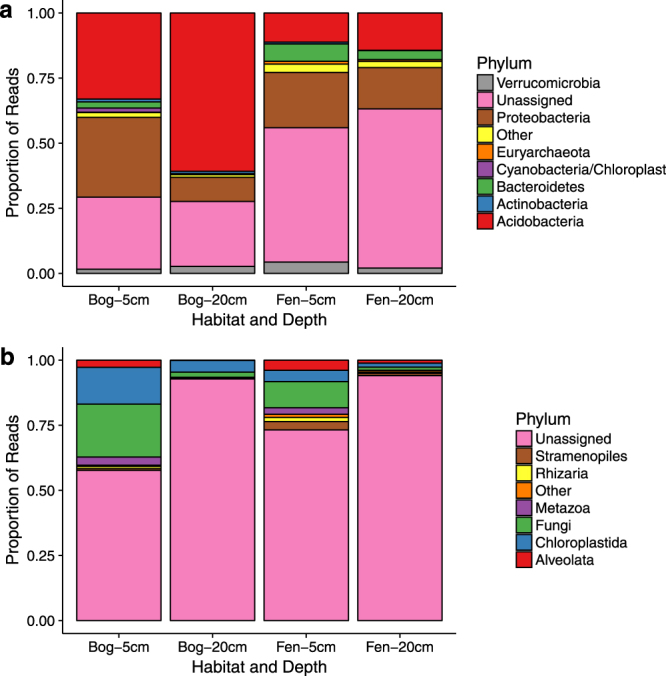
Taxonomic assignment of abundant phyla of (**a**) sequenced reads within the 16S rRNA gene dataset and (**b**) sequenced reads within the 18S rRNA gene dataset. To remove confounding effects of time point and treatment, only data from time point 2 (pre-drought) are shown. Only phyla making up >1% of the community are shown. In each case, assignment was carried out in utax within of usearch v8.12^33^, and assignments with a confidence value of less than 0.85 were classed as unassigned.

Seven prokaryotic phyla and six eukaryotic phyla each made up >1% of reads in at least one habitat and depth (Fig. 3a,b). *Acidobacteria* and *Proteobacteria* contributed by far the highest proportions of prokaryotic reads in both the bog and the fen: Acidobacteria contributed 47% of reads in the bog but only 13% in the fen, while Proteobacteria contributed 20% of reads in the bog and 19% in the fen. However, a large proportion of prokaryotic OTUs could not be assigned to phylum level at the requisite utax confidence level of 0.85, with fen communities containing a higher proportion of ‘Unassigned’ OTUs than bog communities.

Within the eukaryotic communities, an even higher proportion of the community could not be assigned. In particular, at 20 cm depth 90% of reads belonged to OTUS which could not be assigned to phylum level at the chosen confidence level (0.85). Amongst OTUs which could be assigned, the highest numbers of reads were contributed by Chloroplastida (green plants; 9% of reads in the bog and 3% in the fen) and Fungi (11% of reads in the bog and 6% in the fen).

Linear mixed effect models were fitted to transformed proportional abundances of reads from the most abundant phyla in order to determine which factors affected phylum-level community composition. Of the seven prokaryotic phyla which made up >1% of the community, all but *Verrucomicrobia* were significantly affected by habitat and depth, and all were significantly affected by the interaction between habitat and depth (Table S3). In particular, *Acidobacteria* made up a higher proportion of reads in the bog and at 5 cm depth; *Proteobacteria* made up the highest proportion of reads in the bog at 5 cm and the lowest in the bog at 20 cm; and *Bacteroidetes* made up the highest proportion of reads in the fen and at 5 cm depth (Fig. 3a). Conversely, three of the six eukaryotic phyla tested were significantly affected by habitat (Alveolata, Stramenopiles, Rhizaria), four were affected by depth (Fungi, Alveolata, Metazoa, Rhizaria), and four were affected by the interaction between habitat and depth (Fungi, Alveolata, Stramenopiles, Rhizaria; Table S4). Reads assigned to phyla Alveolata, Rhizaria and Stramenopiles were all more abundant in the fen than the bog. Reads assigned to Fungi, Alveolata and Rhizaria were each more abundant at 5 cm than 20 cm.

### Effect of Drought and Environmental Variables on Microbial Communities

**Figure 4:**
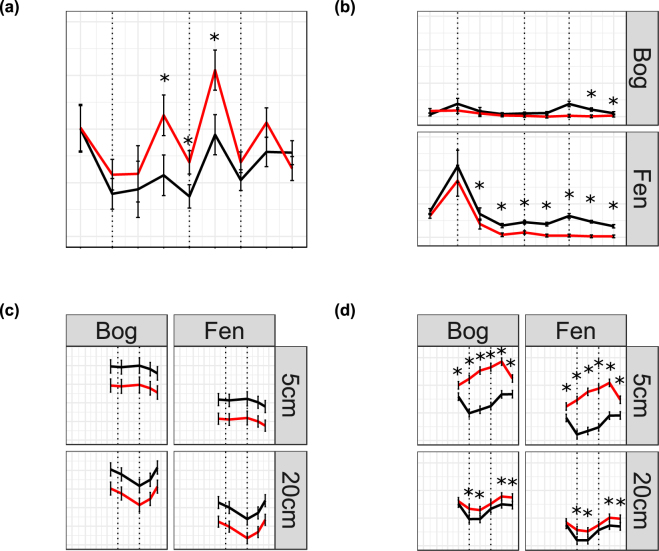
Mean fluxes of (**a**) CO_2_ and (**b**) CH_4_ (mg m^2^ h^−1^), (**c**) concentration of dissolved organic carbon (DOC; mg L^−1^) and (**d**) redox potential (mV) in droughted and control mesocosm cores. Significant differences between the two treatments are marked with *. There was no significant effect of habitat on CO_2_ flux, so means were calculated across both habitats. Error bars show standard errors. Dotted lines on plots (**a**) and (**b**) represent transition between four stages of water table manipulation: pre-drought, drying, minimum water table, and rewetting (in that order); sampling of DOC commenced later and so dotted lines represent transition between drying, minimum water table, and rewetting.

**Figure 5:**
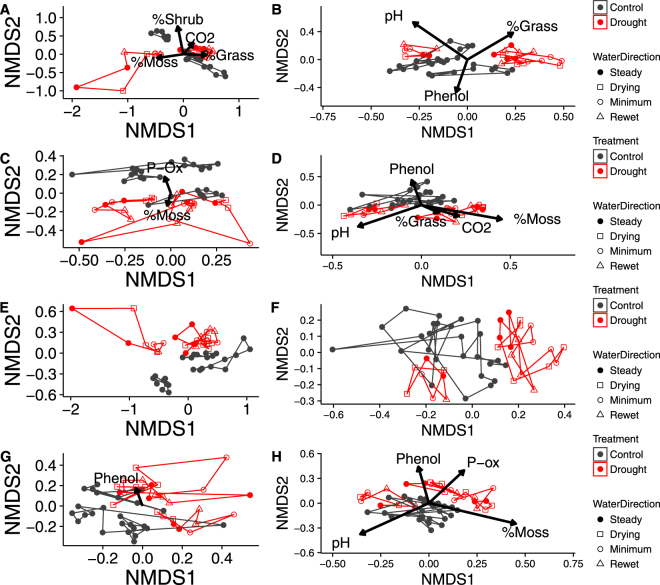
NMDS ordination based on sequencing rRNA genes from of prokaryotic (**A–D**) and eukaryotic (**E–H**) communities within each of the four habitat-depth combinations sampled: (**A,E**) Bog-5 cm, (**B,F**) Fen-5 cm, (**C,G**) Bog-20 cm, and (**D,H**) Fen-20 cm. All samples taken from a given core are connected to form polygons. Arrows depict the results of ‘envfit’ analyses. OTUs assigned to the following phyla were excluded from the eukaryotic dataset prior to analysis: Holozoa, Metazoa, Chloroplastida and ‘Unassigned’.

Under drought conditions and during rewetting, treated mesocosm cores had significantly higher redox potentials and significantly lower water content than control mesocosm cores (Fig. 4d; Fig. S2; Table S5). Carbon dioxide fluxes rose significantly during drought but returned to control levels during rewetting, while methane fluxes fell and remained suppressed throughout the rewetting period (Fig. 4a,b; Table S6). The concentration of dissolved organic carbon (DOC) was significantly lower in fen mesocosm cores than in bog mesocosm cores, and was lower in droughted mesocosm cores (pre-drought measurements of DOC concentration were not taken; Fig. 4c; Table S6). However, there was also an unexpected rise in the water content of the peat between the first two sampling time points (Fig. S2). There was a significant effect of treatment on bacterial ARISA fingerprinting profiles in the bog at both depths and in the fen at 20 cm (Table S7), while the effect of treatment on the fungal community was only significant in the fen at 5 cm. There was a significant two-way interaction between time point and treatment on fungal communities in the fen at 20 cm. In addition, prokaryotic communities at 20 cm in both habitats changed significantly between sampling time points (Table S7) and on fungal communities in the bog at both depths and in the fen at 20 cm (Table S7). However, sequencing of 16S and 18S rRNA genes indicated that there was no effect of the drought-rewetting treatment on overall community composition. NMDS ordination of these communities indicated that the mesocosm core from which samples were taken had a stronger effect on community composition than time point or treatment (Fig. 5). PERMANOVA tests confirmed this observation: while community composition was significantly different between treatments, neither time point nor the interaction effect had a significant effect (Table S8) and therefore the treatment effect observed in sequencing data was likely due to pre-existing differences between the mesocosm cores assigned to each treatment (Fig. 5). Conversely, the effect of core was strongly significant in all habitats and depths and for both markers (Table S9). Application of envfit confirmed differences in microbial communities between mesocosm cores, and also found significant correlations between vegetation and the prokaryotic community (Table 1; Fig. 5). Prokaryotic community composition was significantly correlated to CO_2_ fluxes in the bog at 5 cm depth and the fen at 20 cm depth (although significance was marginal in the latter case), while methane fluxes were not significantly correlated to community composition (Table 1). Fewer significant correlations existed between environmental variables and the community composition of microbial eukaryotes, although there was a weak correlation between eukaryotic community composition and the concentration of phenolic compounds in both habitats at 20 cm depth (Table 1).

**Table 1:** Results of ‘envfit’ applied to ordination of microbial communities within each habitat-depth subset.

Marker	Variable	Bog- 5 cm		Bog- 20 cm		Fen- 5 cm		Fen-20 cm	
		R^2^	p	R^2^	p	R^2^	p	R^2^	p
16S rRNA gene	Core	0.71	0.001**	0.27	0.002**	0.07	0.2	0.14	0.04*
	pH	0.02	0.6	0.03	0.5	0.36	0.001**	0.12	0.045
	Phenol	<0.01	0.9	0.03	0.5	0.22	0.005**	0.35	0.001**
	P-Ox	0.14	0.024*	0.17	0.01*	0.02	0.6	0.05	0.3
	%Moss	0.23	0.002**	0.41	0.001**	0.08	0.1	0.23	0.002**
	%Grasses	0.17	0.017*	0.30	0.001**	0.20	0.006**	0.50	0.001**
	%Shrubs	0.59	0.001**	0.11	0.052	NA	NA	NA	NA
	CO_2_	0.12	0.048*	0.11	0.058	0.02	0.6	0.15	0.02*
	CH_4_	0.04	0.3	0.0174	0.6	0.04	0.4	0.07	0.2
18S rRNA gene	Core	0.04	0.4	<0.01	0.8	0.05	0.3	0.17	0.008**
	pH	<0.01	1	<0.01	0.9	0.01	0.7	0.27	0.001**
	Phenol	0.11	0.07	0.2	0.005**	0.10	0.06	0.20	0.006**
	P-Ox	0.05	0.3	0.04	0.4	0.05	0.3	0.19	0.009**
	%Moss	<0.01	0.8	0.01	0.7	0.08	0.1	0.27	0.001
	%Grasses	0.02	0.7	0.05	0.3	<0.01	0.9	0.03	0.5
	%Shrubs	0.05	0.3	0.08	0.1	NA	NA	NA	NA
	CO_2_	0.03	0.4	0.01	0.8	0.04	0.4	0.08	0.10
	CH_4_	<0.01	0.8	0.05	0.3	0.05	0.3	<0.01	0.9

Significant p-values are denoted by *(p < 0.05), **(p < 0.01), and ***(p < 0.001). OTUs assigned to the following phyla were excluded from the 18 S rRNA dataset prior to analysis: Holozoa, Metazoa, Chloroplastida and ‘NA’. Phenol = concentration of phenolic compounds; P-Ox = phenol oxidase activity; %Moss = percentage cover of mosses; %Grasses = percentage cover of graminoids; %Shrubs = percentage cover of shrubs; CO_2_ = carbon dioxide flux; CH_4_ = methane flux.

**Figure 6:**
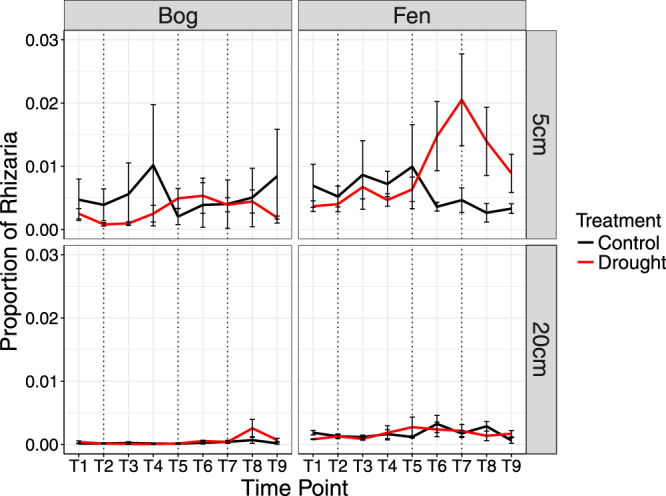
Mean proportion of sequenced 18S rRNA reads belonging to OTUs that were assigned to phylum Rhizaria. Error bars represent standard errors. Dotted lines represent transition between four stages of water table manipulation: pre-drought, drying, minimum water table, and rewetting (in that order).

None of the seven most abundant prokaryotic phyla showed significant changes in relative abundance in response to drought (Table S3). Of the six most eukaryotic phyla, only the relative abundance of Rhizaria was significantly affected by drought, showing an increase in abundance when the water table reached its minimum in the fen at 5 cm depth before falling again during rewetting (interaction between time point and treatment: F_8,177_ = 2.6, *P* = 0.009; Fig. 6; Table S4).

Following abundance filtering of all OTUs, linear mixed effect models were fitted in order to detect OTUs which were significantly affected by the interaction between time point and treatment (hereafter ‘drought-affected OTUs’). Drought-affected OTUs are summarised in Table 2, and full details given in Tables S10–S13 and Figs S3–S6. Briefly, far more drought-affected OTUs were detected in the fen at 5 cm than in any other habitat and depth; in the fen at 5 cm, 37 prokaryotic OTUs and 7 eukaryotic OTUs showed significant changes in relative abundance during drought. Conversely, the number of drought-affected prokaryotic OTUs in other habitats and depths ranged from 2–5 OTUs, while the number of drought affected eukaryotic OTUs ranged from 1–3. NMDS ordination of only drought-affected OTUs confirmed that the effect of drought was most consistent in the fen at 5 cm (Fig. S7).

**Table 2:** Summary of the number and taxonomic affiliation of significantly drought-affected OTUs in sequencing datasets from each habitat and at each depth.

Marker	Habitat	Positive Effect	Negative Effect
16S rRNA gene	Bog-5 cm	Proteobacteria (1); Acidobacteria (2); Bacteroidetes (1)	None
	Bog-20 cm	Acidobacteria (2)	None
	Fen-5 cm	Acidobacteria (1); Bacteroidetes (2); Proteobacteria (11); Unassigned Bacteria (3)	Pacearchaeota (1); Bacteroidetes (12); Firmicutes (1); Proteobacteria (4); Unassigned Bacteria (2)
	Fen-20 cm	Acidobacteria (1); Unassigned Bacteria (1)	Unassigned Bacteria (3)
18S rRNA gene	Bog-5 cm	Rhizaria (2); Unassigned Eukaryote (1)	None
	Bog-20 cm	None	None
	Fen-5 cm	Alveolata (1); Nematoda (1); Rhizaria (1); Unassigned Eukaryote (2)	Unassigned Eukaryote (2)
	Fen-20 cm	Strameopiles (1)	None

Drought-affected OTUs shown were significantly affected by the treatment: time point interaction effect at a p-value of <0.05 prior to the application of corrections for multiple comparisons. Only taxonomic annotations with a utax confidence value of >0.85 are included, with annotations at lower confidence values classed as ‘unassigned’.

Amongst drought-affected OTUs in the fen at 5 cm, the phyla *Proteobacteria* and *Bacteroidetes* were overrepresented relative to their abundance in the dataset as a whole: *Proteobacteria* made up 27% of the overall community and 41% of drought-affected OTUs, while *Bacteroidetes* made up only 7% of the overall community but 39% of drought-affected OTUs. The majority of the drought-affected OTUs which were assigned to *Bacteroidetes* showed a negative response to drought while the majority of those assigned to *Proteobacteria* responded positively, although there were exceptions to this pattern. Few OTUs could be assigned to genus level, but negatively drought-affected OTUs included likely members of genera *Paludibacter* and *Geobacter* while positively drought-affected OTUs included members of genera *Massalia*, *Duganella* and *Caulobacter*. Eukaryotic drought-affected OTUs in the fen at 5 cm contained members of the Alveolata, Rhizaria and Nematoda, as well as four OTUs which could not be assigned at phylum level (Table 2).

Very few drought-affected OTUs occurred in the other habitats. From the 16S rRNA gene dataset, there were five drought-affected OTUs in the fen at 20 cm depth, four in the bog at 5 cm and two in the bog at 20 cm depth. Amongst these, *Acidobacteria* and Unassigned *Bacteria* were the most common taxonomic assignments (Table 2).

## Discussion

While differences in microbial community composition between habitats and depths were detected in analyses based on both ARISA fingerprinting and amplicon sequencing data, the effect of habitat and depth was much stronger when community analysis was based on sequencing data (Fig. 2). The greater resolution in SSU rRNA sequencing data likely results from the limitations of ARISA fingerprinting, which is based on intraspecies differences in the length of the intergenic spacer region of ribosomal rRNA genes. However, in highly diverse environments such as soils, multiple species can share the same intergenic spacer length^31^, reducing the resolution of this technique.

The phylum-level composition of microbial communities in both habitats was similar to previous studies of peat soils^34–36^, suggesting that the composition of peatland communities is conserved across geographically disparate regions, at least at the level of phylum. Relative abundances of all abundant bacterial phyla were significantly affected by both habitat and depth, while only a subset of eukaryotic phyla exhibited demonstrable differences in community composition between habitats and depths. However, phyla containing microbial eukaryotes (Fungi, Stramenopiles, Rhizaria and Alveolata) were more strongly affected by habitat and depth than were macrofaunal phyla, likely because the methods used were not designed to detect variations in the abundance of macrofaunal organisms. Additionally, the large proportion of eukaryotic reads belonging to OTUs which could not be annotated to phylum level likely made differences in abundance more difficult to detect. The strong effect of habitat on the relative abundance of many phyla is unsurprising given that almost all measured environmental variables differed between the two habitats; in comparison to the fen mesocosm cores, bog cores had lower mean pH values and redox potentials, but much higher concentrations of DOC.

Within each habitat and depth, there were significant differences in the community composition of the mesocosm cores, potentially linked to differences in environmental variables between different cores. In particular, the percentage cover of different plant functional groups was significantly correlated to microbial community composition in several cases, as were the concentration of phenolic compounds and the pH of the peat. Plants are an important driver of microbial communities and are able to influence the rhizosphere microbiome directly, for example *via* root exudates^37^. Alternatively, plant communities can act as more effective indicators of soil chemistry over longer time periods, compared to insights derived from a single snapshot in time^38^.

As expected, the drought treatment led to a rise in redox potential and a corresponding release of carbon dioxide while both methane flux and the concentration of DOC fell, corresponding to the results of previous studies^21, 23^. However, unlike previous studies, carbon dioxide fluxes in in droughted mesocosm cores immediately returned to similar levels as observed in control cores when rewetting began, despite the fact that the redox potential remained elevated. The fall in carbon dioxide flux as the water table rises may result from carbon dioxide dissolving in the porewater rather than being released at the surface of the peat; the concentration of dissolved inorganic carbon (DIC) increases rapidly on rewetting^39^, suggesting the potential for porewater to absorb the gases released by microbial metabolism. Alternatively, carbon dioxide release due to increased respiration by autotrophs during drought cannot be ruled out; in some cases, root respiration increases following aeration of peat^39^. Unexpectedly, the water content of the peat rose between the first and second time points in all habitats and at all depths (Fig. S2). The reasons for this rise are unclear as the mesocosm cores were transferred to bins of water within hours of collection, with small holes drilled for water exchange. However, the mesocosm cores in the current experiment were larger than those used in previous studies^40^, creating a potential mechanism for less efficient water exchange between cores and the surrounding water.

Despite the clear effect of drought and rewetting on carbon cycling, the effect of the drought-rewet treatment on microbial community composition was weak and overshadowed by differences between mesocosm cores. ARISA fingerprinting suggested a significant, albeit weak, effect of drought within certain depths and habitats, but there was no corresponding effect in the sequence-based analysis. This discrepancy may have arisen as a result of differences in the lengths of amplicons measured by each method: ARISA amplicons were 165–1,580 bp long, while sequenced rRNA amplicons were 300–350 bp. In freshwater lakes, seasonal changes in community composition derived from analysis of invertebrate environmental DNA have been more rapidly detected when analysing smaller amplicons^41^, as the size distribution of DNA becomes more skewed towards smaller fragments over time^42^.

The weak response of microbial communities to drought and rewetting in both datasets suggests that the increased carbon dioxide flux observed during drought was not mediated by changes in microbial community composition. In addition, CO_2_ fluxes were only significantly correlated to prokaryotic community composition in two of the four possible combinations of habitat and depth (Table 1), and in both cases the correlation was weak (Fig. 5). However, it is possible that members of the microbial community changed in activity rather than abundance, or that genuine community changes were obscured by DNA belonging to dormant or dead organisms^43, 44^. Although not feasible in the present study, metatranscriptomic analyses would further clarify the relative contributions of shifts in the active versus the overall community to drought^45^. While metatranscriptomic analysis has yet to be applied to temperate peatlands, in permafrost peatlands metatranscriptomic analysis gives subtly different results to metagenomics, and so it is likely that differences exist between the active community and the DNA present in soil^46^. In addition, awareness of the role played by rare species in community function and response to environmental change has recently begun to increase^47, 48^; due to the difficulties in separating genuinely rare OTUs from erroneous reads, rare OTUs were not the focus of this study, but it is possible that future studies could gain new insights by focusing on the rare portion of the microbial biosphere in peat ecosystems.

While amplicon sequencing suggested that drought and rewetting did not affect overall community composition, there were nonetheless indications that individual groups of micro-organisms responded to the treatment. In particular, phylum Rhizaria (a phylum of protists) made up a significantly higher proportion of the community in the fen at 5 cm at minimum water table (Fig. 6). The response of Rhizaria to drought is of potential interest, as protists may play important roles in mediating the response of environmental processes to environmental change. For example, grazing by ciliates may determine the rate of change in bacterial biomass under warming conditions^49^ while a fall in the abundance of mixotrophic testate amoeba led to a rise in peatland carbon dioxide emissions following warming^50^. Rhizaria also play an important role in the export of carbon from marine planktonic systems^51^. The role played by protists (especially Rhizaria) in the context of bottom up and top down controls in the carbon cycle of droughted peatlands therefore merits further study.

Testing for significant effects of the drought-rewet treatment on individual prokaryotic OTUs revealed that the relative abundance of a number of OTUs changed relative to control conditions during drought and/or rewetting, particularly in the fen at 5 cm depth. A large proportion of ‘drought-affected OTUs’ in the fen at 5 cm depth belonged to *Bacteroidetes* and *Proteobacteria*. Notably, both of these phyla have been previously identified as containing a high proportion of non-dormant cells when compared to other bacterial phyla^44^, potentially meaning that they more rapidly respond to environmental change by increasing or decreasing in abundance rather than activity. Only two negatively drought-affected OTUs could be assigned to genus level: one of these belonged to genus *Paludibacter*, the sole described member of which is an obligately anaerobic fermenter^52^, and the other to *Geobacter*, a genus of metal-reducers. Therefore, a number of obligate anaerobes may fall in abundance in the active layer of fens following drought. Patterns were more difficult to detect amongst the positively drought-affected OTUs, many of which belonged to the *Proteobacteria*, a diverse phylum containing a broad range of functional categories^53^. Intriguingly, two positively drought-affected OTUs were affiliated with taxa that are commonly associated with petroleum-contaminated soils: genus *Caulobacter* and family *Sphingomonadaceae* ^54, 55^. Both taxa contain aerobic bacteria, prompting speculation that aeration during drought may allow proliferation of bacteria involved in aerobic degradation of organic matter. However, it should be noted that few were significant following the application of corrections for multiple comparisons and thus this analysis should be viewed as a hypothesis-generating rather than a confirmatory study.

Collectively, the current study highlights an array of important insights into the microbial mechanisms underpinning the drought-driven release of carbon from globally important peat ecosystems. The replicated design and enhanced taxonomic resolution afforded by the marker gene analyses demonstrated marked heterogeneity between putatively similar experimental cores. Furthermore, the study suggests that drought-driven changes in carbon fluxes in peatland ecosystems are not associated with large-scale community changes, and thus raises the possibility that these changes may be caused by shifts in the activity rather than the composition of the microbial community or may be a result of small shifts in beta diversity which have large effects on community function. We predict that future combinations of metagenomic and metatranscriptomic analyses will yield further insights to complement existing theories and highlight biogeochemical mechanisms that could be targeted to enhance carbon retention in globally important peat ecosystems.

## Materials and Methods

### Collection of Mesocosm Cores and Experimental Design

Mesocosm cores were collected from two sites representing typical temperate bog and fen habitats. Fen cores were extracted from Cors Erddreiniog, a low-lying fen in mid-Anglesey, North Wales, UK (grid reference SH461826), which is designated a Special Area of Conservation and represents a nationally important area of alkaline and calcareous fen habitat^56^. Bog cores were taken from Marchlyn Mawr (NVC classification M6 [*Carex echinata* – *Sphagnum recurvum/auriculatum* mire]^57^), on the outskirts of Snowdonia National park (grid reference SH610625). Marchlyn Mawr was chosen because of its proximity to important drinking water reservoirs.

Peat ‘mesocosm cores’ were collected in lengths of PVC pipe (each 20 cm in diameter and 35 cm in length), following a protocol adapted from that of Freeman, Lock and Reynolds^40^. After collection, mesocosm cores were kept in a controlled temperature room at 8–10 °C for the duration of the experiment and lit by fluorescent daylight tubes (mean PAR: 305.4 μmol m^−2^ sec^−1^) on a 16:8 hour day-night cycle. Cores were placed in bins which were filled to the level of the peat surface with artificial rainwater for bog cores and artificial groundwater for fen cores, with holes drilled near the base of each core to allow water exchange with the surrounding water. The composition of the rainwater followed a standard recipe^58^, while the groundwater was produced following a custom recipe that emulated the chemical composition of groundwater at Cors Erddreiniog according to earlier measurements (Table S14).

Within each habitat, five of the ten mesocosm cores were randomly assigned to the drought-rewet treatment while the remaining five acted as controls. The water table in the control cores was level with the surface of the peat throughout, mimicking field conditions, while the water table in drought cores at each sampling time point is described in Table S15 and in Fig. 1. Briefly, the first two time points were simulated as ‘pre-drought’, during which the water table in each mesocosm core was level with the peat surface. Following the pre-drought, the water table in treatment cores was gradually lowered over nine weeks, kept stable at 20 cm below the peat surface for six weeks, and rewetted over six weeks (Table S15). The length and intensity of the drought treatment was based on a natural drought which occurred in 2006 in the Cerrig-yr-Wyn catchment in mid-Wales^23^. Additionally, all mesocosm cores were allowed to acclimatise in the controlled temperature room for approximately one month prior to the first sampling time point.

### Sample Collection and DNA Extraction

Soil and gas samples were collected at three week intervals (Table S15). Gas samples were taken between 10am and noon following methods previously used to analyse carbon fluxes from peat mesocosm cores^23^. Briefly, a sealed headspace was placed over each mesocosm core. A 20 cm^3^ gas sample was removed from the headspace at 0, 15, 30, 60 and 120 minutes and injected into an evacuated 12 ml glass vial (Labco Medical Supplies). Gas samples were analysed on a Varian 450-GC fitted with a flame ionisation detector (FID) and a methaniser. At each time point, the machine was calibrated using three gas mixtures of known concentration obtained from Scientific and Technical Gases Ltd (Newcastle under Lyme, Staffordshire, UK). In each case, a linear regression line was calculated between time and carbon dioxide concentration and the slope of the regression was taken as the average flux value.

Subsequently, six gram samples of peat were collected at 5 cm (chosen to correspond to the most biogeochemically active layer of the peat) and 20 cm (chosen to correspond to minimum water table) below the peat surface. Immediately after the removal of soil samples, the redox potential of the peat was measured using a redox probe with an Ag/AgCl reference electrode in 3 M KCl. To adjust the value obtained to the ‘true’ value (i.e. that which would have been obtained using a standard hydrogen electrode), a correction factor of +207 was added prior to further analysis^59^. The holes from which peat samples were taken were immediately plugged to prevent water loss and destruction of the peat structure. The size of the cores was sufficient that each sampling event removed only a small proportion of the total material, and no subsistence of mesocosm cores was observed over the course of the experiment.

The soil samples were homogenised thoroughly using flame-sterilised tools before DNA was extracted from a 0.25 g subsample using a MoBio PowerSoil kit (Cambio, Cambridge), following manufacturer’s instructions. Following preliminary tests, DNA extracted with the MoBio PowerSoil kit was found to contain lower levels of PCR inhibitors than alternative methods. DNA was eluted into 100 µL sterile Tris-EDTA buffer (10 mM Tris, 1 mM EDTA, pH 7.6) and stored at −80 °C prior to further analysis. Samples were further purified using a MoBio PowerClean kit following manufacturer’s instructions, as purification was found to result in more consistent PCR amplification during downstream molecular biological manipulation.

Percentage water content of peat was measured by weighing a subsample of peat before and after drying at 108 °C for 48 hours. Phenol oxidase activity was assayed using the phenolic amino acid L-3,4- dihydroxy phenylalanine (L-DOPA) as a substrate, as described in detail by Dunn *et al*.^60^. The concentration of phenolic compounds was assayed using Folin-Ciocalteu Reagent^61^: briefly, a 1 cm^3^ subsample of peat was taken using a cut-off syringe and weighed. Water-soluble phenolics were extracted by homogenising the peat subsample with 9 ml of water before centrifuging the resulting slurry. 250 μl of supernatant was added to each of three wells of a clear microplate and baseline absorbance measured prior to addition of 12.5 μl Folin-Ciocalteu reagent and 37.5 μl filtered sodium carbonate solution (200 mg l^−1^). Samples were mixed, incubated at room temperature for 90 minutes, and absorbance measured at 750 nm. A calibration curve was produced using dilutions of phenol solution in the range of 0–10 mg l^−1^. A pH meter was inserted into peat slurry (1 g peat: 9 ml water) in order to measure the pH of the peat. DOC measurement was carried out using a Thermalox TOC/TN analyser equipped with a non-dispersive infrared CO_2_ detector.

### ARISA Fingerprinting

Automated ribosomal intergenic spacer analysis (ARISA) is a community fingerprinting technique enabling rapid and low-cost estimation of diversity within a microbial community. The method involves amplifying the intergenic spacer region of microbial ribosomal DNA and analysing the length of the obtained amplicons. The length of the intergenic spacer region is very variable, and amplicons of different sizes are therefore expected to represent separate species or strains^31^. Although all cores were used in biogeochemical analyses (N = 5), this was not possible for nucleic acid-based analyses, and so within each combination of time point and treatment a subset of three of these mesocosm cores were selected for ARISA fingerprinting and all downstream molecular genetic work. Within each chosen core, ARISA fingerprinting was carried out on samples taken from both depths and all nine time points, giving a total of 216 samples for this part of the analysis.

The primers employed for ARISA of bacterial communities were ITSF (5′-GTCGTAACAAGGTAGCCGTA-3′) and ITSReub (5′-GCCAAGGCATCCACC-3′), which have been shown to outperform other commonly used ARISA primers^62^. As there was no existing comparison of primer pairs for ARISA of fungal communities, selected primers were tested using Primer Prospector software^63^. Based on this comparison, a combination of ITS1WH (5′-TCCGTAGGTGAACCTGCGG-3′) and ITS4 (5′-TCCTCCGCTTATTGATATGC-3′) was selected.

Each ARISA PCR reaction contained 9.45 µl of nuclease-free water, 12.5 µl of PCR Master Mix (Promega), 1 µl of each primer (10 µM), 0.05 µl molecular grade bovine serum albumin (1 mg/ml, Thermo Scientific) and 1 µl template DNA (diluted to 10 ng/µl) to give a final volume of 25 µl. For ARISA fingerprinting of bacterial communities, thermal cycling parameters were 95 °C for 2 minutes for initial denaturation, followed by thirty cycles of 95 °C for one minute (denaturation), 52 °C for 45 seconds (annealing), 72 °C for 1.5 minutes (extension), and a final extension period of five minutes. An annealing temperature of 54.2 °C was used for the fungal ARISA primers, with all other steps in the PCR program identical to those for the bacterial communities. PCR amplicon lengths were measured on a Qiaxcel Advanced (Qiagen), using a Qiaxcel High Resolution kit and method OM1200 (recommended by the manufacturer for amplicon lengths between 0.5 and 1.5 kbp).

### Sequencing of 16S and 18S rRNA Genes

As with ARISA fingerprinting, sequencing of rRNA genes could only be carried out for samples taken from a subset of mesocosm cores: for consistency, the same three mesocosm cores were chosen for sequencing as for ARISA fingerprinting. Library preparation, sequencing of 16S and 18S rRNA genes and initial quality control was carried out by the Earth Microbiome Project^64^ (http://www.earthmicrobiome.org/) according to standard protocols. Briefly, the V4 region of the 16S rRNA gene was amplified using primers 515f (5′-GTGCCAGCMGCCGCGGTAA-3′) and 806r (5′-GGACTACHVGGGTWTCTAAT-3′)^65^, which amplify both bacterial and archaeal sequences, and the V9 region of the 18S rDNA gene was amplified using Illumina_Euk_1391f (5′-GTACACACCGCCCGTC-3′) and Illumina_EukBr (5′-TGATCCTTCTGCAGGTTCACCTAC-3′)^66^. Sequencing was carried out on an Illumina HiSeq in rapid run mode, giving paired-end reads of 150 bp in length. Quality control and demultiplexing was carried out in QIITA (http://qiita.microbio.me/), a QIIME-based repository and analysis platform for “-omics” data, and was equivalent to quality control in QIIME using default parameters.

### Sequence Processing and operational taxonomic unit (OTU) Clustering

Following removal of poor quality reads by the Earth Microbiome Project, further quality control and OTU clustering was carried out in VSEARCH^67^, a method which has been proven to output high quality OTUs^68^, followed by taxonomic assignment using USEARCH v8.12^33^. VSEARCH was run on the HPC Wales system. Identical reads were merged and *de novo* chimera prediction was carried out using UCHIME, as implemented in VSEARCH, with default parameters. Next, chimeras were manually removed and OTUs were clustered within VSEARCH at 97% similarity, and an OTU table suitable for downstream analysis was generated using the script ‘uc2otutab.py’ (http://drive5.com/python/uc2otutab_py.html). Taxonomy was assigned to each OTU using the ‘utax’ command in USEARCH v8.12^33^. Taxonomy was assigned against the provided UTAX reference data for 16S rRNA genes, which is based on RDP training set v15^69^, and against the SILVA database v111 for 18S rRNA genes^70^.

Where large differences in read numbers exist between samples and differences between treatments are subtle, rarefaction has been shown to perform outperform other methods of normalization prior to clustering analyses (e.g. NMDS ordination)^71^. Thus, prior to further analysis, read numbers were standardised in all samples using the ‘rrarefy’ command from the ‘VEGAN’ package^72^. Samples in the 16S rRNA gene dataset were standardised to contain 70,000 reads each and samples in the 18S rRNA gene dataset were standardised to contain 20,000 reads each. The thresholds used for standardisation were chosen to include the majority of samples, but exclude samples where sequencing had failed. Samples which contained fewer reads than these thresholds were removed from the dataset: 10 samples were removed from the 16S rRNA gene dataset and 9 from the 18S rRNA gene dataset as they did not contain the requisite number of reads for the read number standardisation step.

### Statistical Analyses

The experimental design gave rise to four independent variables: habitat, depth, treatment and time point. To test for significant effects of these variables on fluxes of carbon dioxide (CO_2_) and methane (CH_4_) and on the concentration of dissolved organic carbon (DOC), linear mixed effect models were fitted using package ‘nlme’ in R^73^. Linear mixed effect models are widely applicable in ecological analyses^74^, and were required in this case to allow for the effect of mesocosm core. Since multiple samples were taken from each mesocosm core, analyses would otherwise have been confounded by temporal pseudoreplication. Model selection was based on the recommendations of Zuur *et al*.^74^. Briefly, models were initially fitted with all main effects (habitat, depth, treatment, and time point) and all two and three-way interactions included. Mesocosm core was included as a random effect. Interaction effects were removed sequentially based on hypothesis testing using a likelihood ratio test until only significant interactions remained (with the exception of the interaction between time point and treatment, which was kept in all models due to the importance of this term to the focal aims of the study).

Community data from ARISA fingerprinting was analysed using the ‘vegan’ package in R^72^. First, fragment sizes were sorted into 5 bp bins and converted to presence-absence data. Next, NMDS ordination was carried out on Jaccard distances across all samples (appropriate for binary (presence-absence) data) followed by PERMANOVA tests.

Following standardisation of sequence numbers across samples, OTU abundance tables were subject to the same multivariate analyses as ARISA fingerprinting data, but based on Bray-Curtis dissimilarity rather than binary Jaccard distances, due to the semi-quantitative information included in this kind of data^75^. In order to focus on the community composition of microbial eukaryotes, all OTUs in the 18S rRNA dataset which were assigned to phyla Holozoa, Chloroplastida and Metazoa (at any confidence level) were excluded from calculations of NMDS ordinations and PERMANOVA tests. Unassigned OTUs were also removed. Results of NMDS ordination were linked to environmental variables using function ‘envfit’ from package ‘VEGAN’^72^: this was done within habitat-depth subsets due to the strong effect of habitat and depth on both microbial community composition and environmental variables. Envfit first calculates the direction of the effect of a given variable: for ‘vectors’ (continuous variables) this is done by calculating the direction of maximum correlation between the variable and the ordination scores, while for ‘factors’ (discrete variables) envfit calculates the average ordination score for each factor level. Next, significance values are calculated for each variable using a permutation test.

To test for significant effects of habitat, depth, treatment and time point on the most abundant phyla (i.e. those which made up >1% of the community), the proportion of each phylum was logit-transformed^76^ and linear mixed effect models were fitted as described above for gas fluxes.

Linear mixed effect models were also used to identify individual OTUs that showed significant responses to drought. Within each habitat-depth combination, OTUs were first filtered to include only those OTUs which were sufficiently abundant (at least 1 read per 1000 reads in one sample) and present in at least 20% of samples. This strict filtering was carried out in order to minimise effects of rare OTUs; the high proportion of rare OTUs in the dataset was considered likely to generate spurious results. Following filtering, the relative abundance of each OTU was logit transformed^76^ and linear mixed effect models were fitted with mesocosm core as a random effect (random intercept model). Benjamini-Hochberg corrections^77^ were calculated to correct for the large number of comparisons. Where a significant effect of the interaction between time point and treatment was found, OTU abundances were carefully scrutinised and cases where the interaction effect was due to outlier effects were removed.

### Data accessibility

 Sequence data is publically archived on the ENA/EBI database (accession number ERP016584) and on QIITA (https://qiita.ucsd.edu/;study ID:10278).

## Electronic supplementary material

Supplementary Tables and Figures
