# Supplementary material for: Subtle shifts in microbial communities occur alongside the release of carbon induced by drought and rewetting in contrasting peatland ecosystems
Source: Sci Rep. 2017 Sep 12;7:11314. doi: 10.1038/s41598-017-11546-w (PMC5595961; doi:10.1038/s41598-017-11546-w)
Supplement: Supplementary file 1 — Supplementary Tables and Figures [file 41598_2017_11546_MOESM1_ESM.doc]

# Subtle shifts in microbial communities occur alongside the release of carbon induced by drought and rewetting in contrasting peatland ecosystems

**Running head:** Drought and peat microbial communities

Caitlin Potter^1,3^, Chris Freeman^1^, Peter N. Golyshin^1^, Gail Ackermann^2^, Nathalie Fenner^1^, James E. McDonald^1^, Abdessalam Ehbair^1,3^, Tim Jones^1^, Lorrie Murphy^1^, and Simon Creer^1*^.

**Table S1** Results of PERMANOVA tests for an effect of habitat and depth on the ARISA fingerprinting profiles of bacteria and fungi. Significant p-values are denoted by * (p < 0.05), ** (p < 0.01), and *** (p < 0.001). Marginal significance (p < 0.1) is denoted by ‘**.**’.

| **Community** | **Variable** | **F** | **d.f.** | **R^2^** | **p** |
| --- | --- | --- | --- | --- | --- |
| **Bacteria** | **Habitat** | 10.0682 | 1,210 | 0.04357 | 0.001** |
|  | **Depth** | 6.3164 | 1,210 | 0.02733 | 0.001** |
|  | **Habitat: Depth** | 4.7193 | 1,210 | 0.02042 | 0.001** |
| **Fungi** | **Habitat** | 7.2602 | 1 | 0.02988 | 0.001** |
|  | **Depth** | 22.3763 | 1 | 0.0921 | 0.001** |
|  | **Habitat: Depth** | 2.331 | 1 | 0.00959 | 0.01* |

**Table S2 PERMANOVA tests of the effect of habitat and depth on the community composition of prokaryotes and eukaryotes as determined by sequencing of 16S and 18S rRNA genes respectively. Significant p-values are denoted by * (p < 0.05), ** (p < 0.01), and *** (p < 0.001). Marginal significance (p < 0.1) is denoted by ‘.’. OTUs assigned to the following phyla were excluded from the 18S rRNA dataset prior to analysis: Holozoa, Metazoa, Chloroplastida and ‘NA’.**

| **Marker** | **Factor** | **df** | **F** | **r^2^** | **p** |
| --- | --- | --- | --- | --- | --- |
| **16S** | **Habitat** | 1 | 228.0 | 0.38 | 0.001** |
|  | **Depth** | 1 | 94.6 | 0.16 | 0.001** |
|  | **Habitat: Depth** | 1 | 82.5 | 0.14 | 0.001** |
| **18S** | **Habitat** | 1 | 25.8 | 0.10 | 0.001** |
|  | **Depth** | 1 | 13.7 | 0.05 | 0.001** |
|  | **Habitat: Depth** | 1 | 11.3 | 0.04 | 0.001** |

**Table S3** Minimal adequate linear mixed effects model with the proportion of reads assigned to each of the most abundant prokaryotic phyla (at a confidence value of 0.85 or above) as the response variable. Non-significant interactions are not shown, with the exception of the interaction between time point and treatment. Significant p-values are denoted by * (p < 0.05), ** (p < 0.01), and *** (p < 0.001). Marginal significance (p < 0.1) is denoted by ‘**.**’. Degrees of freedom reported are reported in the form: d.f._denominator_, d.f._numerator_.

| **Phylum** | **Factor** | **df** | **F** | **p** |
| --- | --- | --- | --- | --- |
| **Acidobacteria** | **Habitat** | 1, 9 | 170.3 | <0.0001*** |
|  | **Depth** | 1, 168 | 402.8 | <0.0001*** |
|  | **Time Point** | 8, 168 | 1.2 | 0.3 |
|  | **Treatment** | 1, 9 | <0.1 | 1 |
|  | **Habitat:Depth** | 1, 168 | 107.0 | <0.0001*** |
|  | **Habitat:Time Point** | 8, 168 | 2.0 | 0.045* |
|  | **Time Point: Treatment** | 8, 168 | 0.3 | 1 |
| **Actinobacteria** | **Habitat** | 1, 8 | 10.2 | 0.01* |
|  | **Depth** | 1, 166 | 28.8 | <0.0001*** |
|  | **Time Point** | 8, 166 | 0.7 | 0.7 |
|  | **Treatment** | 1, 8 | 8.3 | 0.02* |
|  | **Habitat: Depth** | 1, 166 | 14.9 | 0.0002*** |
|  | **Habitat: Treatment** | 1, 8 | 5.6 | 0.045* |
|  | **Depth: Treatment** | 1, 166 | 34.1 | <0.0001*** |
|  | **Habitat: Depth: Treatment** | 1, 166 | 27.7 | <0.0001*** |
| **Bacteroidetes** | **Habitat** | 1,8 | 147.4 | <0.0001*** |
|  | **Depth** | 1, 174 | 464.3 | <0.0001*** |
|  | **Time Point** | 8, 174 | 0.5 | 0.9 |
|  | **Treatment** | 1, 8 | 3.0 | 0.1 |
|  | **Habitat: Depth** | 1, 174 | 130.9 | <0.0001*** |
|  | **Habitat: Treatment** | 1, 8 | 5.1 | 0.055 |
|  | **Depth: Treatment** | 1, 174 | 8.7 | 0.004** |
|  | **Time Point: Treatment** | 8, 174 | 0.8 | 0.6 |
|  | **Habitat:Depth:Treatment** | 1, 174 | 7.6 | 0.007** |
| **Euryarchaeota** | **Habitat** | 1, 8 | 7.9 | 0.02* |
|  | **Depth** | 1, 174 | 19.6 | <0.0001*** |
|  | **Time Point** | 8, 174 | 1.0 | 0.4 |
|  | **Treatment** | 1, 8 | 6.8 | 0.03* |
|  | **Habitat: Depth** | 1, 174 | 41.7 | <0.0001*** |
|  | **Depth: Treatment** | 1, 174 | 60.8 | <0.0001*** |
|  | **Time Point: Treatment** | 8, 174 | 0.7 | 0.7 |
|  | **Habitat: Depth: Treatment** | 1, 174 | 28.1 | <0.0001*** |
| **Proteobacteria** | **Habitat** | 1, 9 | 76.8 | <0.0001*** |
|  | **Depth** | 1, 176 | 350.0 | <0.0001*** |
|  | **Time Point** | 8, 176 | 0.3 | 1 |
|  | **Treatment** | 1, 9 | 0.3 | 0.6 |
|  | **Habitat: Depth** | 1, 176 | 78.2 | <0.0001*** |
|  | **Time Point: Treatment** | 8, 176 | 1.0 | 0.4 |
| **Cyanobacteria/**  **Chloroplast** | **Habitat** | 1, 9 | 7.7 | 0.02* |
|  | **Depth** | 1, 168 | 293.4 | <0.0001*** |
|  | **Time Point** | 8, 168 | 2.2 | 0.03* |
|  | **Treatment** | 1, 9 | 2.4 | 0.2 |
|  | **Habitat:Depth** | 1, 168 | 40.5 | <0.0001*** |
|  | **Habitat: Time Point** | 8, 168 | 2.9 | 0.005** |
|  | **Time Point: Treatment** | 8, 168 | 0.5 | 0.8 |
| **Verrucomicrobia** | **Habitat** | 1, 9 | 1.2 | 0.3 |
|  | **Depth** | 1, 173 | 3.4 | 0.07. |
|  | **Time Point** | 8, 173 | 0.9 | 0.5 |
|  | **Treatment** | 1, 9 | 2.6 | 0.1 |
|  | **Habitat: Depth** | 1, 173 | 52.6 | <0.0001*** |
|  | **Depth: Treatment** | 1, 173 | 8.6 | 0.004** |
|  | **Time Point: Treatment** | 8, 173 | 0.6 | 0.8 |
|  | **Habitat: Depth: Treatment** | 2, 173 | 4.5 | 0.01* |

**Table S4:** Minimal adequate linear mixed effects model with the proportion of reads assigned to the most abundant eukaryotic phyla (at a confidence value of 0.85 or higher) as the response variable. Non-significant interactions are not shown, with the exception of the interaction between time point and treatment. Significant p-values are denoted by * (p < 0.05), ** (p < 0.01), and *** (p < 0.001). Marginal significance (p < 0.1) is denoted by ‘**.**’. Degrees of freedom reported are reported in the form: d.f._denominator_, d.f._numerator_.

| **Phylum** | **Factor** | **df** | **F** | **p** |
| --- | --- | --- | --- | --- |
| **Fungi** | **Habitat** | 1, 8 | 3.4 | 0.1 |
|  | **Depth** | 1, 175 | 149.0 | <0.0001*** |
|  | **Time Point** | 8, 175 | 0.9 | 0.5 |
|  | **Treatment** | 1, 8 | 0.1 | 0.8 |
|  | **Habitat: Depth** | 1, 175 | 5.7 | 0.02* |
|  | **Habitat: Treatment** | 1, 8 | 4.2 | 0.07. |
|  | **Depth: Treatment** | 1, 175 | 14.9 | 0.0002*** |
|  | **Time Point: Treatment** | 8, 175 | 1.0 | 0.5 |
|  | **Habitat: Depth: Treatment** | 1, 175 | 17.0 | 0.0001*** |
| **Chloroplastida** | **Habitat** | 1, 8 | 2.4 | 0.2 |
|  | **Depth** | 1, 175 | 0.6 | 0.4 |
|  | **Time Point** | 8, 175 | 0.5 | 0.8 |
|  | **Treatment** | 1, 8 | <0.1 | 0.8 |
|  | **Depth: Treatment** | 1, 175 | 18.9 | <0.0001*** |
|  | **Time Point: Treatment** | 8, 175 | 0.7 | 0.7 |
|  | **Habitat: Depth: Treatment** | 1, 175 | 24.4 | <0.0001*** |
| **Alveolata** | **Habitat** | 1, 9 | 100.1 | <0.0001*** |
|  | **Depth** | 1, 177 | 330.7 | <0.0001*** |
|  | **Time Point** | 8, 177 | 0.9 | 0.6 |
|  | **Treatment** | 1, 9 | 1.5 | 0.3 |
|  | **Habitat: Depth** | 1, 177 | 58.8 | <0.0001*** |
|  | **Time Point: Treatment** | 8, 177 | 0.4 | 0.9 |
| **Stramenopiles** | **Habitat** | 1, 8 | 23.1 | 0.001** |
|  | **Depth** | 1, 175 | 2.9 | 0.09. |
|  | **Time Point** | 8, 175 | 0.9 | 0.5 |
|  | **Treatment** | 1, 8 | 2.4 | 0.2 |
|  | **Habitat: Depth** | 1, 175 | 15.5 | 0.0001*** |
|  | **Depth: Treatment** | 1, 175 | 9.7 | 0.002*** |
|  | **Time Point: Treatment** | 8, 175 | 1.1 | 0.4 |
|  | **Habitat: Depth: Treatment** | 1, 175 | 8.6 | 0.004** |
| **Metazoa** | **Habitat** | 1, 9 | 2.7 | 0.1 |
|  | **Depth** | 1, 178 | 439.2 | <0.0001*** |
|  | **Time Point** | 8, 178 | 0.8 | 0.6 |
|  | **Treatment** | 1, 9 | 0.1 | 0.8 |
|  | **Time Point: Treatment** | 8, 178 | 0.9 | 0.5 |
| **Rhizaria** | **Habitat** | 1, 9 | 24.1 | 0.0008*** |
|  | **Depth** | 1, 177 | 217.1 | <0.0001*** |
|  | **Time Point** | 8, 177 | 0.4 | 0.9 |
|  | **Treatment** | 1, 9 | 0.1 | 0.8 |
|  | **Habitat: Depth** | 1, 177 | 18.5 | <0.0001*** |
|  | **Time Point: Treatment** | 8, 177 | 2.6 | 0.009** |

**Table S5 Minimal adequate linear mixed effects model with water content (arcsine-transformed proportion) and redox potential as as the response variable. Significant p-values are denoted by * (p < 0.05), ** (p < 0.01), and *** (p < 0.001). Marginal significance (p < 0.1) is denoted by ‘.’. Degrees of freedom reported are reported in the form: d.f._denominator_, d.f._numerator_.**

| **Variable** | **Effect** | **F** | **d.f.** | **p** |
| --- | --- | --- | --- | --- |
| **Water Content** | Habitat | 26.0 | 1,17 | <0.001*** |
|  | Depth | 43.5 | 1,306 | <0.001*** |
|  | Time Point | 4.7 | 8,306 | <0.001*** |
|  | Treatment | 2.8 | 1,17 | 0.110 |
|  | Time Point: Treatment | 2.01 | 8,306 | 0.04* |
|  | Time Point: Habitat | 2.8 | 8,306 | 0.006** |
|  | Time Point: Depth | 4.6 | 8,306 | <0.001*** |
|  | Treatment: Depth | 13.3 | 1,306 | <0.001*** |
| **Redox** | Habitat | 179.1 | 1,17 | <0.001*** |
|  | Treatment | 6.4 | 1,17 | 0.022* |
|  | Time Point | 3.7 | 5,198 | 0.034* |
|  | Time Point: Treatment | 5.0 | 5,198 | <0.001*** |
|  | Depth: Time Point: Treatment | 3.1 | 5,198 | 0.011* |

**Table S6** Results of minimal adequate linear mixed-effect models with fluxes of carbon dioxide and methane as response variables. Significant p-values are denoted by * (p < 0.05), ** (p < 0.01), and *** (p < 0.001). Marginal significance (p < 0.1) is denoted by ‘**.**’. Degrees of freedom reported are reported in the form: d.f._denominator_, d.f._numerator_.

| **Variable** | **Factor** | **F** | **d.f.** | **p** |
| --- | --- | --- | --- | --- |
| **CO_2_ Flux** | Time Point | 2.1 | 4,144 | 0.04* |
|  | Treatment | <0.01 | 1,18 | 1.0 |
|  | Time Point: Treatment | 3.8 | 4,144 | <0.001*** |
| **CH_4_ Flux** | Habitat | 51.6 | 1,16 | <0.001*** |
|  | Time Point | 4.7 | 8,136 | <0.001*** |
|  | Time Point: Habitat | 9.3 | 8,136 | 0.002** |
|  | Habitat: Treatment | 7.0 | 1,16 | 0.02* |
|  | Time Point: Treatment | 6.7 | 8,13 | <0.001*** |
| **DOC** | Date | 5.0 | 5,117 | 0.0002** |
|  | Habitat | 29.5 | 1,17 | <0.001*** |
|  | Depth | 0.2 | 1,177 | 0.6 |
|  | Treatment | 10.5 | 1,17 | 0.0047** |
|  | Date:Depth | 2.4 | 5,177 | 0.0376* |

**Table S7** Results of PERMANOVA in which bacterial and fungal ARISA fingerprinting profiles were the dependent variables while time, treatment and the time:treatment interaction term were independent variables. Significant p-values are denoted by * (p < 0.05), ** (p < 0.01), and *** (p < 0.001). Marginal significance (p < 0.1) is denoted by ‘.’. PERMANOVA was carried out separately for each combination of habitat and depth and for both marker genes.

| **Community** | **Data Subset** | **Variable** | **F** | **d.f.** | **R^2^** | **p** |
| --- | --- | --- | --- | --- | --- | --- |
| **Bacteria** | **Bog-5cm** | **Treatment** | 1.5 | 1 | 0.03 | 0.001** |
|  |  | **Time Point** | 1.1 | 8 | 0.16 | 0.1 |
|  |  | **Treatment: Time Point** | 0.9 | 8 | 0.13 | 0.9 |
|  | **Bog-20cm** | **Treatment** | 1.5 | 1 | 0.03 | 0.04* |
|  |  | **Time Point** | 1.3 | 8 | 0.19 | 0.004** |
|  |  | **Treatment: Time Point** | 1 | 8 | 0.14 | 0.6 |
|  | **Fen-5cm** | **Treatment** | 1.4 | 1 | 0.03 | 0.1 |
|  |  | **Time Point** | 1.1 | 8 | 0.16 | 0.3 |
|  |  | **Treatment: Time Point** | 0.9 | 8 | 0.14 | 0.8 |
|  | **Fen-20cm** | **Treatment** | 1.7 | 1 | 0.03 | 0.02* |
|  |  | **Time Point** | 1.3 | 8 | 0.18 | 0.01* |
|  |  | **Treatment: Time Point** | 1.1 | 8 | 0.13 | 0.2 |
| **Fungi** | **Bog-5cm** | **Treatment** | 1.0 | 1 | 0.02 | 0.4 |
|  |  | **Time Point** | 2.0 | 8 | 0.26 | 0.001** |
|  |  | **Treatment: Time Point** | 1.0 | 8 | 0.13 | 0.4 |
|  | **Bog-20cm** | **Treatment** | 0.9 | 1 | 0.01 | 0.6 |
|  |  | **Time Point** | 1.8 | 8 | 0.24 | 0.001** |
|  |  | **Treatment: Time Point** | 1.1 | 8 | 0.14 | 0.3 |
|  | **Fen-5cm** | **Treatment** | 1.9 | 1 | 0.03 | 0.04* |
|  |  | **Time Point** | 1.5 | 8 | 0.20 | 0.01* |
|  |  | **Treatment: Time Point** | 1.1 | 8 | 0.15 | 0.3 |
|  | **Fen-20cm** | **Treatment** | 2.2 | 1 | 0.04 | 0.09. |
|  |  | **Time Point** | 1.0 | 8 | 0.14 | 0.4 |
|  |  | **Treatment: Time Point** | 1.5 | 8 | 0.20 | 0.09. |

**Table S8** Results of PERMANOVA tests for the effect of time point, treatment and the time point: treatment interaction term on the community composition of prokaryotes (16S rRNA genes) and eukaryotes (18S rRNA genes), based on sequencing data. Significant p-values are denoted by * (p < 0.05), ** (p < 0.01), and *** (p < 0.001). Marginal significance (p < 0.1) is denoted by ‘.’. PERMANOVA was carried out separately for each combination of habitat and depth and for both marker genes. OTUs assigned to the following phyla were excluded from the 18S rRNA dataset prior to analysis: Holozoa, Metazoa, Chloroplastida and ‘NA’.

| **Marker** | **Habitat-Depth** | **Factor** | **df** | **F** | **R^2^** | **p** |
| --- | --- | --- | --- | --- | --- | --- |
| **16S rRNA gene** | **Bog-5cm** | **Time Point** | 8 | 0.6 | 0.58 | 1 |
|  |  | **Treatment** | 1 | 3.2 | 0.07 | 0.002** |
|  |  | **Time Point: Treatment** | 8 | 0.4 | 0.07 | 1 |
|  | **Fen- 5cm** | **Time Point** | 8 | 0.8 | 0.13 | 0.9 |
|  |  | **Treatment** | 1 | 5.3 | 5.35 | 0.001** |
|  |  | **Time Point: Treatment** | 8 | 0.7 | 0.68 | 1 |
|  | **Bog- 20cm** | **Time Point** | 8 | 0.4 | 0.07 | 1 |
|  |  | **Treatment** | 1 | 8.3 | 0.17 | 0.001** |
|  |  | **Time Point: Treatment** | 8 | 0.3 | 0.04 | 1 |
|  | **Fen- 20cm** | **Time Point** | 8 | 0.6 | 0.11 | 1 |
|  |  | **Treatment** | 1 | 5.6 | 0.12 | 0.001** |
|  |  | **Time Point: Treatment** | 8 | 0.5 | 0.09 | 1 |
| **18S rRNA gene** | **Bog-5cm** | **Time Point** | 8 | 0.1 | 0.13 | 1 |
|  |  | **Treatment** | 1 | 3.6 | 0.07 | 0.001** |
|  |  | **Time Point: Treatment** | 8 | 0.8 | 0.12 | 1 |
|  | **Fen- 5cm** | **Time Point** | 8 | 0.8 | 0.13 | 1 |
|  |  | **Treatment** | 1 | 2.4 | 0.47 | 0.001** |
|  |  | **Time Point: Treatment** | 8 | 0.8 | 0.13 | 1 |
|  | **Bog- 20cm** | **Time Point** | 8 | 1.0 | 0.15 | 1 |
|  |  | **Treatment** | 1 | 1.5 | 0.03 | 0.001** |
|  |  | **Time Point: Treatment** | 8 | 0.9 | 0.15 | 1 |
|  | **Fen- 20cm** | **Time Point** | 8 | 0.9 | 0.15 | 1 |
|  |  | **Treatment** | 1 | 2.3 | 0.05 | 0.001** |

**Table S9** Results of PERMANOVA tests for an effect of mesocosm core on the community composition of prokaryotes (16S rRNA genes) and eukaryotes (18S rRNA genes), based on sequencing data. Tests were carried out separately for each combination of habitat and depth and for both marker genes. Significant p-values are denoted by * (p < 0.05), ** (p < 0.01), and *** (p < 0.001). OTUs assigned to the following phyla were excluded from the 18S rRNA dataset prior to analysis: Holozoa, Metazoa, Chloroplastida and ‘NA’.

| **Marker** | **Habitat-Depth** | **df** | **F** | **r^2^** | **p** |
| --- | --- | --- | --- | --- | --- |
| **16S rRNA gene** | **Bog- 5cm** | 5 | 12.6 | 0.58 | 0.001** |
|  | **Fen- 5cm** | 5 | 6.2 | 0.41 | 0.001** |
|  | **Bog- 20cm** | 5 | 21.3 | 0.69 | 0.001** |
|  | **Fen- 20cm** | 5 | 8.1 | 0.48 | 0.001** |
| **18S rRNA gene** | **Bog- 5cm** | 5 | 4.5 | 0.33 | 0.001** |
|  | **Fen- 5cm** | 5 | 2.7 | 0.23 | 0.001** |
|  | **Bog- 20cm** | 5 | 1.6 | 0.15 | 0.001** |
|  | **Fen- 20cm** | 5 | 2.2 | 0.20 | 0.001** |

**Table S10 Linear mixed-effects models with logit-transformed abundances of prokaryotes (16S rRNA) and eukaryotes (18S rRNA) in the fen at 5cm as response variables. Adjusted p-values (Benjami-Hochberg correction) are shown in column ‘p.adj’. Only OTUs with an (unadjusted) p-value of <0.05 for the time point: treatment interaction term are shown. In addition, significant results were manually filtered by plotting abundance in each mesocosm core against time, and cases where significance was due to one or two extreme values were excluded. Significant adjusted p-values are denoted by * (p < 0.05), ** (p < 0.01), and *** (p < 0.001). Marginal significance (p < 0.1) is denoted by ‘.’. Taxonomy was assigned using the RDP Classifier web server, as this was found to classify OTUs with more confidence than utax. Column ‘graph’ refers to the graph on Fig. S4 which corresponds to each OTU.**

|  | **OTU** | **F** | **d.f.** | **p** | **p.adj** | **Graph** | **Taxonomy** |
| --- | --- | --- | --- | --- | --- | --- | --- |
| **16S** | OTU_503 | 8.2 | 8,28 | <0.0001 | 0.008** | A | Bacteria; Proteobacteria; Beta-Proteobacteria |
|  | OTU_469 | 6.7 | 8,28 | <0.0001 | 0.046* | B | Bacteria; Proteobacteria; Gamma-Proteobacteria |
|  | OTU_871 | 6.0 | 8,28 | 0.0002 | 0.1 | C | Bacteria; Proteobacteria; Beta-Proteobacteria; Burkholderiales; Oxalobacteraceae; *Massalia* |
|  | OTU_699 | 5.4 | 8,28 | 0.004 | 0.3 | D | Bacteria; Bacteroidetes |
|  | OTU_226 | 5.2 | 8,28 | 0.0005 | 0.4 | E | Bacteria; Bacteroidetes |
|  | OTU_744 | 5.1 | 8,28 | 0.0005 | 0.4 | F | Bacteria; Proteobacteria; Alpha-Proteobactera; Rhizobiales |
|  | OTU_473 | 5.0 | 8,28 | 0.0007 | 0.5 | G | Bacteria; Bacteroidetes |
|  | OTU_1031 | 4.9 | 8,28 | 0.0007 | 0.5 | H | Bacteria; Bacteroidetes |
|  | OTU_1076 | 4.9 | 8,28 | 0.0007 | 0.5 | I | Bacteria; Bacteroidetes |
|  | OTU_845 | 4.2 | 8,28 | 0.002 | 1 | J | Bacteria |
|  | OTU_943 | 4.1 | 8,28 | 0.002 | 1 | K | Bacteria; Bacteroidetes; Bacteroidia; Bacteroidales; Porphyromandaceae; *Paludibacter* |
|  | OTU_20783 | 4.0 | 8,28 | 0.002 | 1 | L | Bacteria; Bacteroidetes |
|  | OTU_13911 | 3.9 | 8,28 | 0.004 | 1 | M | Bacteria; Proteobacteria |
|  | OTU_290 | 3.8 | 8,28 | 0.004 | 1 | N | Bacteria |
|  | OTU_695 | 3.7 | 8,28 | 0.004 | 1 | O | Bacteria; Bacteroidetes; Spingobacteriia; Sphingobacteriales |
|  | OTU_381 | 3.7 | 8,28 | 0.005 | 1 | P | Bacteria; Proteobacteria; Beta-Proteobacteria; Burkholderiales; Oxalobacteraceae; *Duganella* |
|  | OTU_204 | 3.6 | 8,28 | 0.005 | 1 | Q | Bacteria |
|  | OTU_2224 | 3.4 | 8,28 | 0.007 | 1 | R | Bacteria |
|  | OTU_932 | 3.2 | 8,28 | 0.010 | 1 | S | Bacteria; Proteobacteria; Delta-Proteobacteria |
|  | OTU_836 | 3.2 | 8,28 | 0.011 | 1 | T | Bacteria; Bacteroidetes |
|  | OTU_1515 | 3.1 | 8,28 | 0.012 | 1 | U | Bacteria; Proteobacteria |
|  | OTU_720 | 3.0 | 8,28 | 0.014 | 1 | V | Bacteria; Proteobacteria; Alpha-Proteobacteria; Caulobacterales; Caulobacteraceae; *Caulobacter* |
|  | OTU_1109 | 3.0 | 8,28 | 0.015 | 1 | W | Bacteria; Proteobacteria |
|  | OTU_515 | 3.0 | 8,28 | 0.015 | 1 | X | Archaea; Pacearchaeota |
|  | OTU_405 | 3.0 | 8,28 | 0.016 | 1 | Y | Bacteria; Bacteroidetes |
|  | OTU_793 | 2.9 | 8,28 | 0.017 | 1 | Z | Bacteria; Bacteroidetes |
|  | OTU_15950 | 2.8 | 8,28 | 0.022 | 1 | AA | Bacteria; Proteobacteria; Delta-Proteobacteria; Desulfuromonadales; Geobacteraceae; *Geobacter* |
|  | OTU_3414 | 2.7 | 8,28 | 0.024 | 1 | AB | Bacteria; Firmicutes; Clostridia; Clostridiales; Ruminococcaceae |
|  | OTU_545 | 2.7 | 8,28 | 0.026 | 1 | AC | Bacteria; Bacteroidetes; Bacteroidia; Bacteroidales |
|  | OTU_2189 | 2.6 | 8,28 | 0.027 | 1 | AD | Bacteria; Bacteroidetes |
|  | OTU_2114 | 2.6 | 8,28 | 0.028 | 1 | AE | Bacteria |
|  | OTU_601 | 2.6 | 8,28 | 0.032 | 1 | AF | Bacteria; Proteobacteria; Delta-Proteobacteria |
|  | OTU_1143 | 2.4 | 8,28 | 0.040 | 1 | AG | Bacteria; Bacteroidetes |
|  | OTU_14319 | 2.3 | 8,28 | 0.045 | 1 | AH | Bacteria; Acidobacteria; Group 6 |
|  | OTU_26696 | 2.3 | 8,28 | 0.046 | 1 | AI | Bacteria; Proteobacteria; Alpha-Proteobacteria; Sphingomonadales; Sphingomonadaceae; Sphingomonas |
|  | OTU_683 | 2.3 | 8,28 | 0.048 | 1 | AJ | Bacteria; Proteobacteria; Alpha-Proteobacteria; Rhizobiales |
|  | OTU_37782 | 2.3 | 8,28 | 0.048 | 1 | AK | Bacteria; Proteobacteria; Beta-Proteobacteria |
| **18S** | OTU_96 | 3.5 | 8,30 | 0.005 | 0.8 | AL | Eukaryota; Rhizaria; Cercozoa |
|  | OTU_389 | 2.8 | 8,30 | 0.02 | 1 | AM | Eukaryota |
|  | OTU_206 | 2.7 | 8,30 | 0.02 | 1 | AN | Eukaryota |
|  | OTU_62 | 2.5 | 8,30 | 0.03 | 1 | AO | Eukaryota; Alveolata |
|  | OTU_262 | 2.4 | 8,30 | 0.04 | 1 | AP | Eukaryota |
|  | OTU_102 | 2.3 | 8,30 | 0.04 | 1 | AQ | Eukaryota |
|  | OTU_51 | 2.3 | 8,30 | 0.05 | 1 | AR | Eukaryota; Metazoa; Nematoda |

**Table S11** Linear mixed-effects models with logit-transformed abundances of prokaryotes (16S rRNA) and eukaryotes (18S rRNA) in the bog at 5cm as response variables**.** Adjusted p-values (Benjami-Hochberg correction) are shown in column ‘p.adj’. Only OTUs with an (unadjusted) p-value of <0.05 for the time point: treatment interaction term are shown. In addition, significant results were manually filtered by plotting abundance in each mesocosm core against time, and cases where significance was due to one or two extreme values were ignored. Significant adjusted p-values are denoted by * (p < 0.05), ** (p < 0.01), and *** (p < 0.001). Marginal significance (p < 0.1) is denoted by ‘**.**’. Taxonomy was assigned using the RDP Classifier web server, as this was found to classify OTUs with more confidence than utax. Column ‘graph’ refers to the graph on Fig. S5 which corresponds to each OTU.

|  | **OTU** | **F** | **d.f.** | **p** | **p.adj** | **Graph** | **Taxonomy** |
| --- | --- | --- | --- | --- | --- | --- | --- |
| **16S** | OTU_748 | 3.7 | 8,30 | <0.0001 | 1 | A | Bacteria; Proteobacteria; Alpha-Proteobacteria; Rhodospirillales; Acetobacteraceae |
|  | OTU_156 | 3.4 | 8,30 | 0.01 | 1 | B | Bacteria; Acidobacteria; Group 6 |
|  | OTU_46137 | 3.1 | 8,30 | 0.01 | 1 | C | Bacteria; Acidobacteria; Group 1 |
|  | OTU_695 | 2.7 | 8,30 | 0.02 | 1 | D | Bacteria; Bacteroidetes; Spingobacteriia, Spingobacteriales |
| **18S** | OTU_189 | 4.9 | 8,30 | 0.00 | 0.06. | E | Eukaryota |
|  | OTU_74 | 3.2 | 8,30 | 0.01 | 0.96 | F | Eukaryota; Rhizaria |
|  | OTU_23322 | 3.1 | 8,30 | 0.01 | 1 | G | Eukaryota; Rhizaria; Cercozoa |

**Table S12** Linear mixed-effects models with logit-transformed abundances of prokaryotes (16S rRNA) and eukaryotes (18S rRNA) in the fen at 20cm as response variables**.** Adjusted p-values (Benjami-Hochberg correction) are shown in column ‘p.adj’. Only OTUs with an (unadjusted) p-value of <0.05 for the time point: treatment interaction term are shown. In addition, significant results were manually filtered by plotting abundance in each mesocosm core against time, and cases where significance was due to one or two extreme values were ignored. Significant adjusted p-values are denoted by * (p < 0.05), ** (p < 0.01), and *** (p < 0.001). Marginal significance (p < 0.1) is denoted by ‘**.**’ Taxonomy was assigned using the RDP Classifier web server, as this was found to classify OTUs with more confidence than utax. Column ‘graph’ refers to the graph on Fig. S6 which corresponds to each OTU.

| **Marker** | **OTU** | **F** | **d.f.** | **p** | **p.adj** | **Graph** | **Taxonomy** |
| --- | --- | --- | --- | --- | --- | --- | --- |
| **16S** | OTU_606 | 3.1 | 8, 28 | 0.01 | 1 | A | Bacteria |
|  | OTU_364 | 2.6 | 8, 28 | 0.03 | 1 | B | Bacteria |
|  | OTU_524 | 2.4 | 8, 28 | 0.04 | 1 | C | Bacteria |
|  | OTU_75 | 2.4 | 8, 28 | 0.04 | 1 | D | Bacteria; Acidobacteria; Group 1 |
|  | OTU_62 | 2.3 | 8, 28 | 0.045 | 1 | E | Bacteria |
| **18S** | OTU_40802 | 3.0 | 8, 28 | 0.01 | 1 | F | Eukaryota; Stramenopiles |

**Table S13** Linear mixed-effects models with logit-transformed abundances of eukaryotes (16S rRNA) and prokaryotes (18S rRNA) in the bog at 20cm as response variables**.** Adjusted p-values (Benjami-Hochberg correction) are shown in column ‘p.adj’. Only OTUs with an (unadjusted) p-value of <0.05 for the time point: treatment interaction term are shown. In addition, significant results were manually filtered by plotting abundance in each mesocosm core against time, and cases where significance was due to one or two extreme values were ignored. Significant adjusted p-values are denoted by * (p < 0.05), ** (p < 0.01), and *** (p < 0.001). Marginal significance (p < 0.1) is denoted by ‘**.**’. Taxonomy was assigned using the RDP Classifier web server, as this was found to classify OTUs with more confidence than utax. Column ‘graph’ refers to the graph on Fig. S7 which corresponds to each OTU.

| **Marker** | **OTU** | **F** | **d.f.** | **p** | **p.adj** | **Graph** | **Taxonomy** |
| --- | --- | --- | --- | --- | --- | --- | --- |
| **16S** | OTU_4221 | 2.9 | 8, 32 | 0.02 | 1 | A | Bacteria; Acidobacteria; Group 1 |
|  | OTU_1152 | 2.8 | 8, 32 | 0.02 | 1 | B | Bacteria; Acidobacteria; Group 1 |

**Table S14 Final concentration of important ions in artificial rainwater and groundwater.** Also shown are measured concentrations of ions from Cors Erddreiniog (N. Menichino, pers. comm.). Unfortunately, it was not possible to exactly match concentrations from the groundwater using available equipment and salts. Concentrations are given in mg l^-1^.

| **Ion** | **Artificial Groundwater** | **Artificial Rainwater** | **Cors E. Groundwater** |
| --- | --- | --- | --- |
| Na^2+^ | 2.4 | 2.5 | - |
| K^+^ | 0.1 | 0.1 | - |
| Ca^2+^ | 0.3 | 5.2 | 124.5 |
| Mg^+^ | 0.7 | 2.9 | 5.5 |
| Cl^-^ | 0.7 | 10.2 | 15.1 |
| SO_4_^-^ | 3.1 | 3.3 | 3.0 |
| NH_4_^+^ | 0.1 | 0.0 | 0.1 |
| NO_3_^-^ | 0.0 | 0.1 | 0.1 |

**Table S15** Depth beneath the surface of the water table in drought cores throughout the experiment. Water table depth was adjusted at twice-weekly intervals by removing water from each bin containing a drought core. Column ‘Sample’ indicates dates at which samples were collected.

| **Date** | **Water Table** | **Sample** | **Water Sample** |
| --- | --- | --- | --- |
| 28/08/2013 | 0 | T1 |  |
| 02/09/2013 | 0 |  |  |
| 09/09/2013 | 0 |  |  |
| 16/09/2013 | 0 |  |  |
| 23/09/2013 | 0 | T2 |  |
| 30/09/2013 | -2.2 |  |  |
| 07/10/2013 | -4.4 |  |  |
| 14/10/2013 | -6.6 | T3 |  |
| 21/10/2013 | -8.8 |  |  |
| 28/10/2013 | -11 |  |  |
| 04/11/2013 | -13.2 | T4 |  |
| 11/11/2013 | -15.4 |  | W1 |
| 18/11/2013 | -17.6 |  |  |
| 25/11/2013 | -20 | T5 |  |
| 02/12/2013 | -20 |  | W2 |
| 09/12/2013 | -20 |  |  |
| 16/12/2013 | -20 | T6 |  |
| 23/12/2013 | -20 |  |  |
| 30/12/2013 | -20 |  |  |
| 06/01/2014 | -17 | T7 | W3 |
| 13/01/2014 | -14 |  |  |
| 20/01/2014 | -11 |  |  |
| 27/01/2014 | -8 | T8 | W4 |
| 03/02/2014 | -5 |  |  |
| 10/02/2014 | -2 |  | W5 |
| 17/02/2014 | 0 | T9 |  |


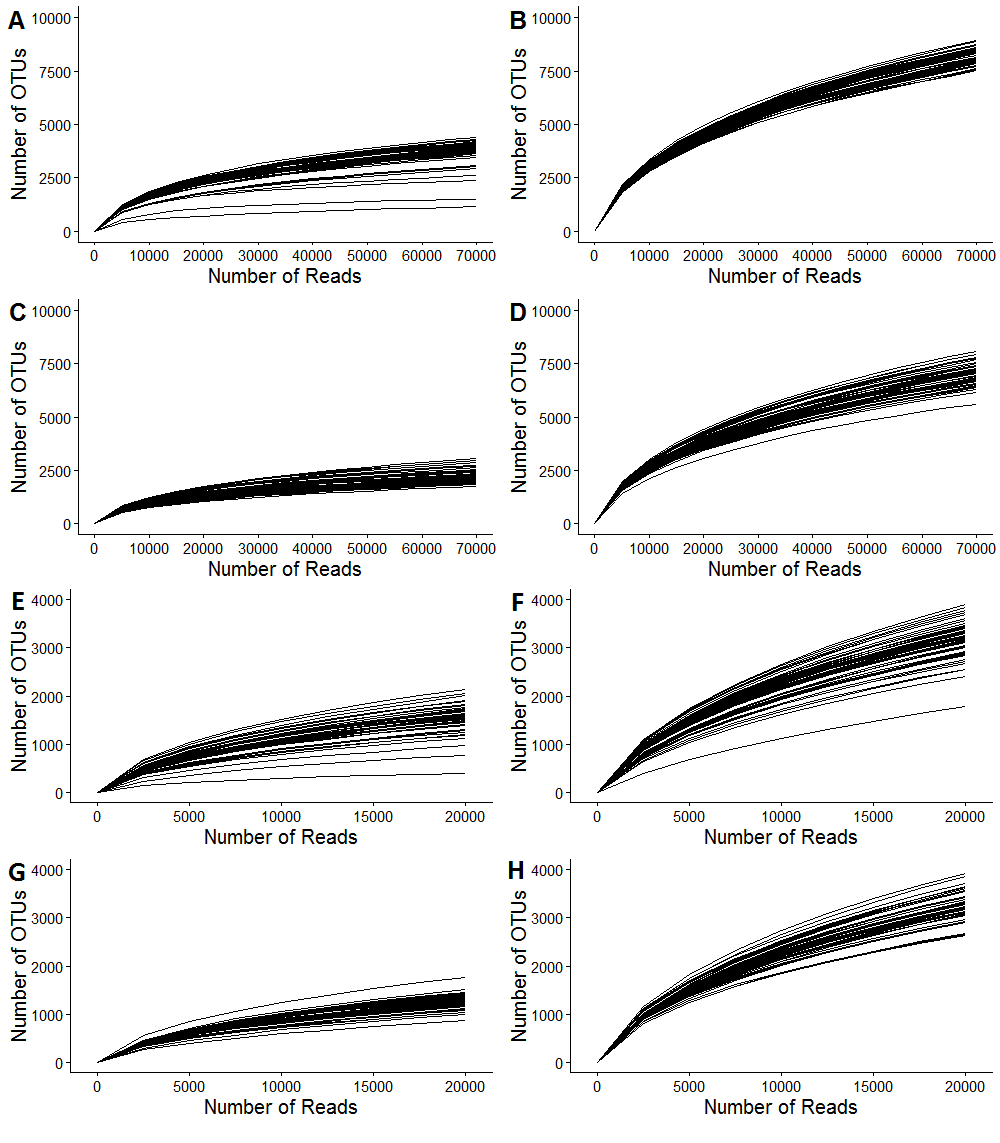


**Fig. S1** Rarefaction analysis of prokaryotic (A-D) and eukaryotic (E-H) communities, with each line representing the results of rarefaction analysis on a single sample. Results are plotted seperately for each habitat-depth combination as follows: A and E, bog-5cm; B and F, fen-5cm; C and G, bog-20cm; D and H, fen-20cm. Rarefaction analysis was carried out after standardisation of read numbers within each sample.


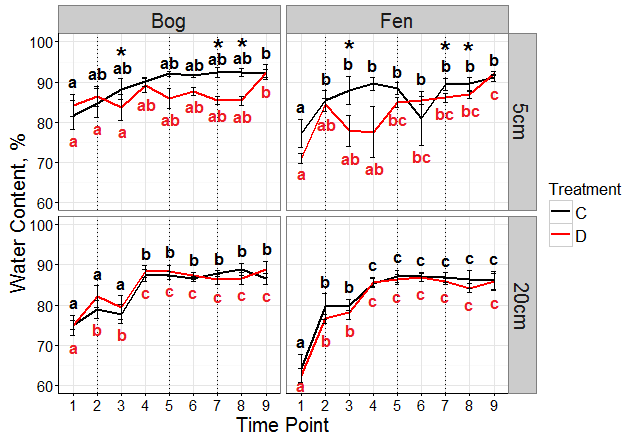


**Fig. S2** Mean water content as a percentage of total wet mass. Significant differences between the two treatments are marked with *, while significant differences between time points are marked with different letters (red = drought, black = control). Error bars show standard errors. Dotted lines represent transition between four stages of water table manipulation: pre-drought, drying, minimum water table, and rewetting (in that order).


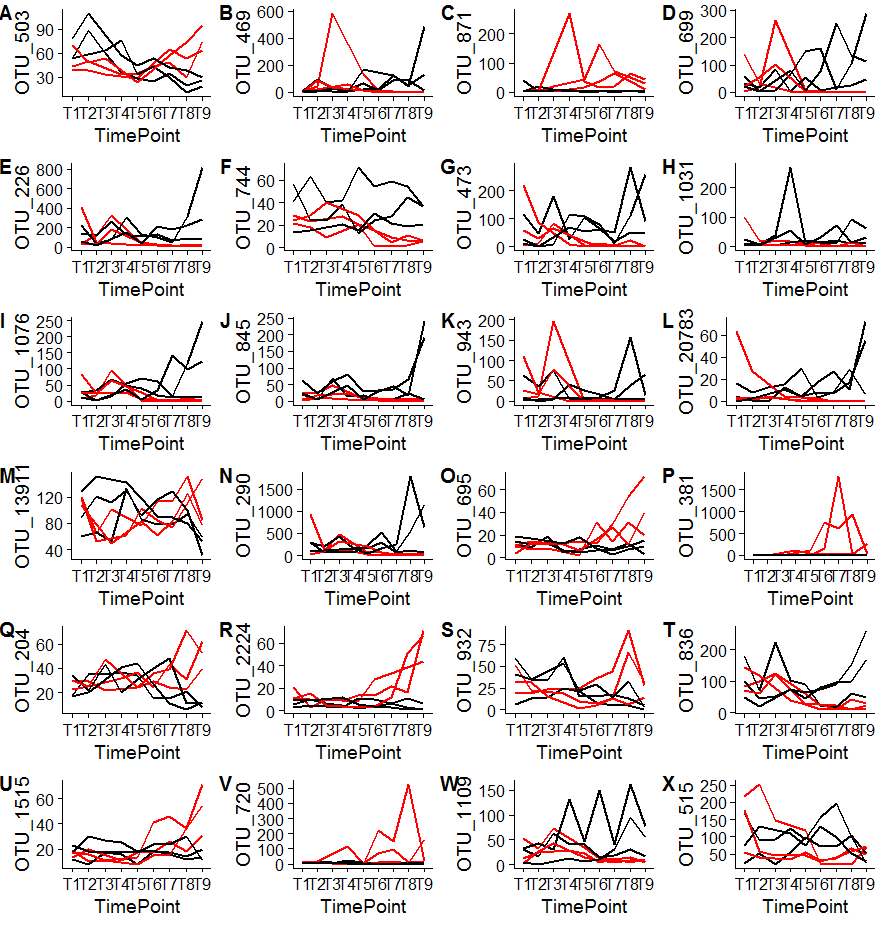


**Fig. S3A** Line plots showing the abundance of OTUs which were found to be significantly affected by time point and treatment in the fen at 5cm. Each fen mesocosm core is plotted individually to show the degree of individual variation in abundance between mesocosm cores: red lines represent droughted cores, and black lines represent control cores. Further information on each OTU is displayed in Table S9.


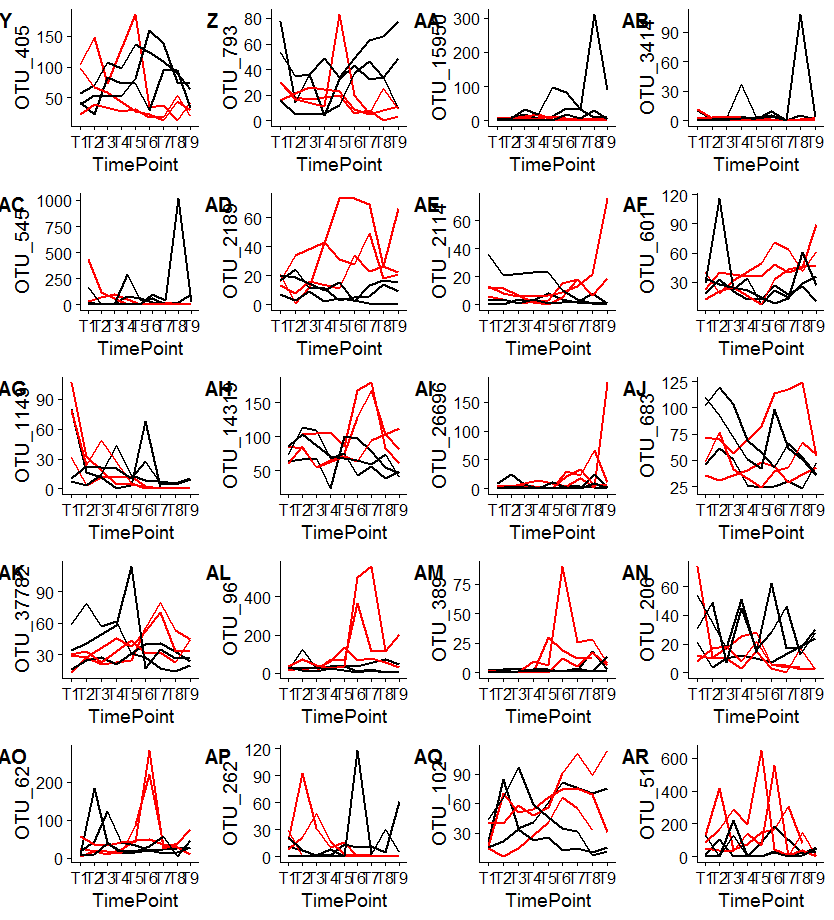


**Fig. S3B (continued from S4A)**


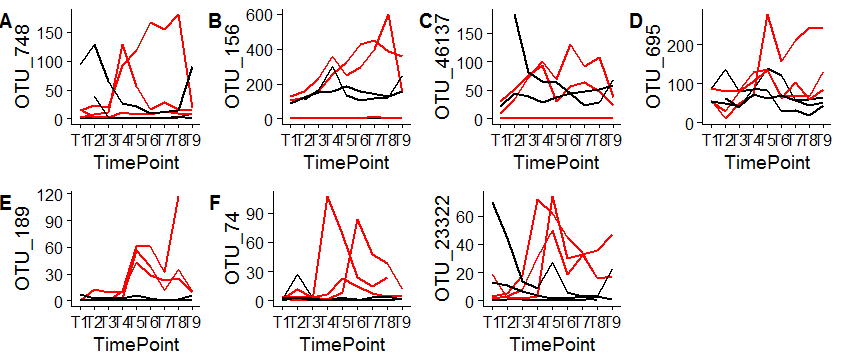


**Fig. S4** Line plots showing the abundance of OTUs which were found to be significantly affected by time point and treatment in the bog at 5cm. Each bog core is plotted individually to show the degree of individual variation in abundances between mesocosm cores: red lines represent droughted cores, and black lines represent control cores. Further information on each OTU is displayed in Table S10.


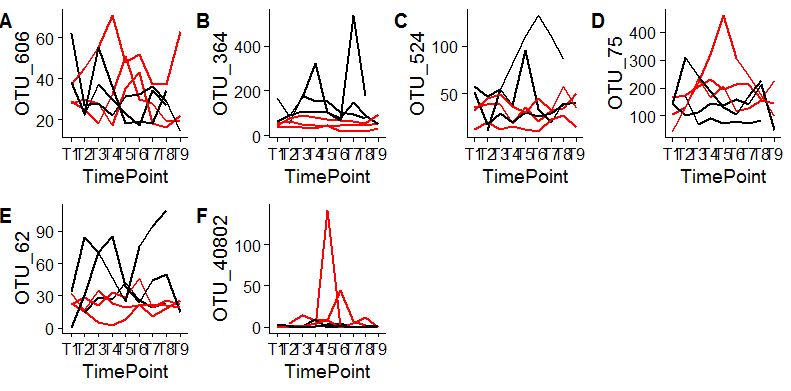


**Fig. S5** Line plots showing the abundance of OTUs which were found to be significantly affected by time point and treatment in the fen at 20cm. Each fen core is plotted individually to show the degree of individual variation in abundances between mesocosm cores: red lines represent droughted cores, and black lines represent control cores. Further information on each OTU is displayed in Table S11.


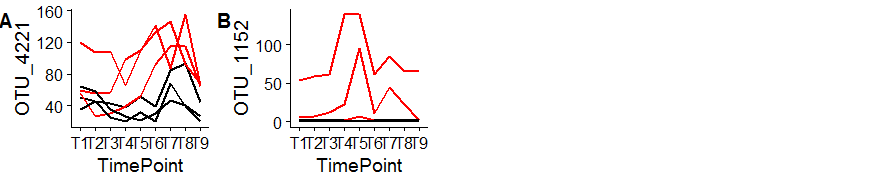


**Fig. S6** Line plots showing the abundance of OTUs which were found to be significantly affected by time point and treatment in the bog at 20cm. Each bog core is plotted individually to show the degree of individual variation in abundances between mesocosm cores: red lines represent droughted cores, and black lines represent control cores. Further information on each OTU is displayed in Table S12.


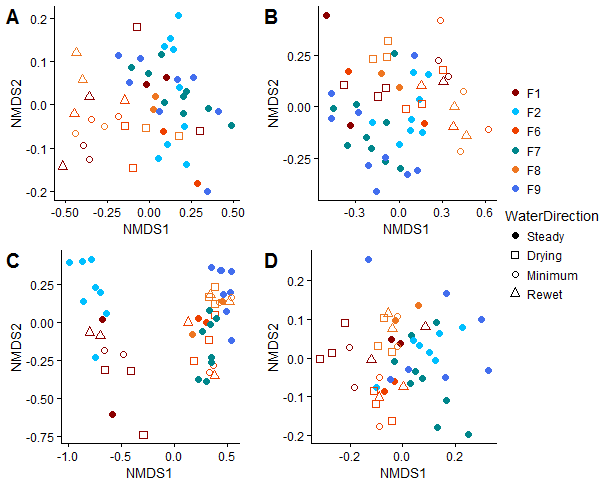
**Fig. S7** NMDS ordination of the drought-affected OTUs- put the full tables and figures in Supplementary and a summary table in the main text. Plots A & B show ordination of drought-affected OTUs from the fen at 5cm, divided into prokaryotes (A) and eukaryotes (B). Plots C & D show drought-affected prokaryotic OTUs in the bog at 5cm (C) and the fen at 20cm (D).
